# Dynamic phosphorylation of TCOF1 controlled by CK2 and PPM1D regulates nucleolar condensation and RNA polymerase I activity

**DOI:** 10.1093/nar/gkag1060

**Published:** 2026-10-30

**Authors:** Natalie Palkova, Matous Palek, Marketa Brodinova, Zuzana Chalupova, Radka Storchova, Jana Jasprova, Pavel Talacko, Dusan Cmarko, Libor Macurek

**Affiliations:** Cancer Cell Biology, Institute of Molecular Genetics of the Czech Academy of Sciences, Prague, CZ-14220, Czech Republic; Cancer Cell Biology, Institute of Molecular Genetics of the Czech Academy of Sciences, Prague, CZ-14220, Czech Republic; Cancer Cell Biology, Institute of Molecular Genetics of the Czech Academy of Sciences, Prague, CZ-14220, Czech Republic; Cancer Cell Biology, Institute of Molecular Genetics of the Czech Academy of Sciences, Prague, CZ-14220, Czech Republic; Cancer Cell Biology, Institute of Molecular Genetics of the Czech Academy of Sciences, Prague, CZ-14220, Czech Republic; Proteomics Core Facility, Faculty of Science, Charles University, Prague, CZ-12800, Czech Republic; Proteomics Core Facility, Faculty of Science, Charles University, Prague, CZ-12800, Czech Republic; Institute of Biology and Medical Genetics, First Faculty of Medicine, Charles University and General University Hospital in Prague, Prague, CZ-12800, Czech Republic; Cancer Cell Biology, Institute of Molecular Genetics of the Czech Academy of Sciences, Prague, CZ-14220, Czech Republic

## Abstract

The nucleolus is a membrane-less organelle essential for ribosomal DNA (rDNA) transcription and ribosome biogenesis, yet the mechanisms regulating its dynamic assembly remain incompletely understood. Here, we identify the phosphatase protein phosphatase magnesium-dependent 1 delta (PPM1D) as a key regulator of the nucleolar architecture via dephosphorylation of the scaffold protein TCOF1. By combining super-resolution microscopy and domain mapping, we show that PPM1D localizes to the nucleolus through a conserved motif at the C-terminus. Phosphoproteomic analysis and structural modeling revealed that PPM1D targets casein kinase 2 (CK2)-phosphorylated sites within the low-complexity repeat domain of TCOF1, which impacts its interaction with RNA polymerase I (Pol I), immobilization in the fibrillar centres, and nucleolar condensation. Disruption of the phosphorylation balance by loss of PPM1D or inhibition of CK2 leads to nucleolar disorganization and reduced rDNA transcription. Finally, we demonstrate that pharmacological inhibition of PPM1D sensitizes cells to RNA Pol I inhibitors, in both p53-dependent and -independent contexts. Together, we uncover a reversible phosphorylation switch that governs TCOF1 dynamics and its nucleolar function, with potential implications for targeting ribosome biogenesis in cancer.

## Graphical Abstract

**Graphical Abstract:**
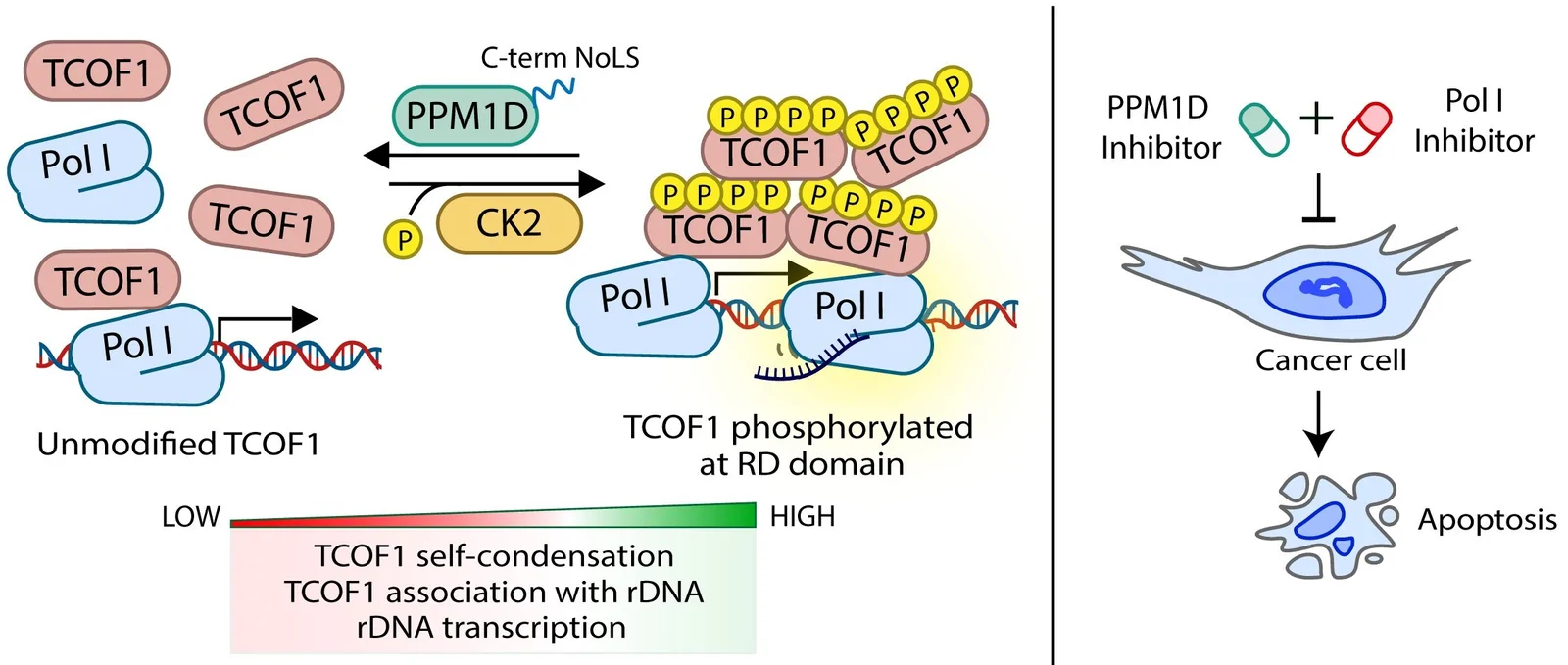

## Introduction

Nucleoli are self-assembling non-membrane nuclear structures formed around clusters of the ribosomal RNA genes (rDNA) located at the acrocentric chromosomes and play an essential role in the biogenesis of the ribosomes that are required for translation of messenger RNA to proteins [1]. This process starts with rDNA transcription at the interface of the fibrillar centers (FCs) and the dense fibrillar component (DFC), followed by processing of rRNA and storage of the immature ribosomes in the granular component (GC) [2]. Nucleolar rDNA is transcribed by RNA polymerase I (Pol I) into the precursor transcript that is processed into the mature 18S, 5.8S, and 28S rRNAs [3–5]. In a multistep process, mature rRNAs assemble with >80 ribosomal proteins and with 5S rRNA transcribed by Pol III outside the nucleolus, jointly forming the 60S and 40S ribosomal subunits. The function of Pol I critically depends on its association with the Selectivity factor 1 (SL1), Upstream-Binding Factor (UBF), and RNA Pol I-specific transcription initiation factor (RRN3) at promoters [6–9]. Importantly, assembly of the Pol I complex is regulated by phosphorylation of several subunits by casein kinase 2 (CK2) and other kinases to correlate rRNA production with the cell cycle, nutrient availability, and stress signaling [10–13]. Phosphorylation of the C-terminal domain of UBF stabilizes its interaction with SL1 and promotes transcription [14]. Regulation of the interaction between RRN3 and the RPA43 subunit of the Pol I is more complex [15, 16]. The non-phosphorylated RRN3 interacts with RPA43, promotes loading of Pol I on promoters, and initiates the transcription [17]. In turn, RRN3 is phosphorylated by CK2 and dissociates from Pol I during the elongation phase of transcription [16, 17]. After dephosphorylation, RRN3 binds another Pol I molecule, allowing a next round of rDNA transcription [17]. Thus, ribosomal biogenesis depends on dynamic changes in phosphorylation of individual RNA Pol I subunits.

Treacle (hereafter referred to as TCOF1) is a nucleolar phosphoprotein mutated in the rare craniofacial disorder Treacher Collins syndrome [18]. TCOF1 binds UBF and Pol I by its C-terminal domain (amino acids 1294–1488) and central repeat domain (RD) (amino acids 526–961), respectively, and recruits them to rDNA promoters [19, 20]. Depletion of TCOF1 leads to dissociation of Pol I, UBF, and DEAD-box RNA helicase DDX21 from the nucleolus and reduction of rDNA transcription [19, 21]. Phosphoproteomic data suggests that TCOF1 is massively modified under basal conditions and following genotoxic stress [22, 23]. In response to DNA damage, CK2- and ATM-dependent modification of TCOF1 promotes re-localization of NBS1 protein to the nucleoli, which leads to suppression of rDNA transcription and translocation of rDNA into nucleolar caps, where it is repaired by homologous recombination [23–25]. During embryogenesis, CK2 phosphorylates the central part of TCOF1 in the neural crest cells and promotes its interaction with the ubiquitin ligase complex CUL3^KBTBD8^, which is required for establishing a correct translational program during neural conversion [26, 27]. Nevertheless, the impact of the constitutive, CK2-mediated phosphorylations of the TCOF1 on its function in ribosome biogenesis has not been systematically investigated.

Several nucleolar proteins, including fibrillarin, nucleophosmin (NPM1), and TCOF1 contain intrinsically disordered regions (IDRs) comprising the charged amino acid repeats that facilitate nucleolar formation by liquid–liquid phase separation (LLPS) [28–30]. For instance, acidic regions in NPM1 form homotypic and heterotypic interactions with the arginine-rich linear motifs of other nucleolar proteins and with rRNA, and promote formation of the biomolecular condensates [31, 32]. Similarly, homotypic interactions of the serine/glutamate-rich low-complexity regions (LCRs) promote TCOF1 self-assembly and are necessary for formation of the FC component of the nucleoli [33, 34].

Protein phosphatase magnesium-dependent 1 delta (PPM1D, also known as PP2C delta or WIP1) is a nuclear Ser/Thr phosphatase implicated in the timely termination of the DNA damage response and inhibition of the tumor suppressor p53, jointly allowing cells to recover from the cell cycle arrest and continue proliferation [35–39]. PPM1D is an oncogene, and its amplification or gain-of-function mutations allow cells to escape the tumor-suppressive barriers represented by programmed cell death or senescence and promote various human cancers, including breast cancer, glioma, and hematological malignancies [40–44]. The inhibition of PPM1D has been proposed as potential pharmacological target in cancers with the wild-type p53 [45, 46]. Proteomic screens suggest that PPM1D functions can reach beyond its canonical role in silencing the DNA damage–induced cell cycle checkpoints [47]; nevertheless, the spectrum of its physiological substrates and its involvement in basal cellular functions still remain largely unexplored.

Here we show that a fraction of PPM1D localizes to the nucleoli, where it counteracts the CK2-mediated phosphorylation of TCOF1. Phosphorylation of TCOF1 LCRs drives its condensation and promotes its immobilization in the FCs. PPM1D modulates dynamic interaction of TCOF1 with RNA Pol I and promotes rDNA transcription. In the absence of PPM1D, transcription of rDNA is reduced, nucleoli are enlarged, and their structure is impaired. Cells lacking PPM1D are highly sensitive to Pol I inhibitors, suggesting that combined inhibition of PPM1D and RNA Pol I might be efficient strategy to kill cancer cells.

## Materials and methods

### Plasmids

A plasmid coding for human EGFP-TCOF1 was a kind gift from Manuel Stucki. The coding sequence was cloned into pSB-Tet-Pur (Addgene ID: 60507, [48]) to enable generation of cell lines with doxycycline-inducible expression. The TCOF1-ΔRD variant was derived by deleting region coding for 83–1170, as already described [33]. DNA fragment coding the TCOF1-SA variant, with all serines in all 17 TCOF1 repeats mutated to alanines, was synthetized by Thermo Fisher and was subcloned into pSB-Tet-Pur backbone. 2xHA-TCOF1 was cloned into NheI and BamHI sites of pEGFP-C1 backbone, leading to removal of EGFP sequence. The pEGFP-C1-PPM1D construct coding for wild-type PPM1D and its deletion variants was already described [49]. To generate cell lines with inducible expression, pEGFP-PPM1D was cloned into pSB-Tet backbone with either puromycin or blasticidine resistance. PPM1D-ΔNoLS was generated by deleting the region coding for 581–589 residues. The coding sequence of all plasmids was confirmed using Sanger sequencing.

### Cell lines

Human hTERT-immortalized RPE1 cells (referred to as RPE), human osteosarcoma U2OS cells, and human breast adenocarcinoma MCF7 cells were grown in high-glucose Dulbecco’s modified Eagle’s medium (DMEM) supplemented with 6% fetal bovine serum (FBS) (Gibco), Penicillin (10 U/ml), and Streptomycin (0.1 mg/ml). All cell lines were regularly tested for mycoplasma contamination using MycoAlert kit (Lonza). To generate cell lines with inducible EGFP-TCOF1 expression, pSB-Tet-Pur plasmids for EGFP-TCOF1 variants were co-transfected with SB100 (1:10 weight ratio) using PEI. Stably transfected cells were selected in medium containing 1.5 µg/ml puromycin for 10 days. RPE-PPM1D-KO and U2OS-PPM1D-KO cells have been already described [50, 51]. To generate cells with inducible expression, RPE-PPM1D-KO cells were co-transfected with pSB-Tet-Blast-PPM1D-WT or -ΔNOLS variant and SB100, and selected using 1.5 µg/ml blasticidin for 2 weeks. Plasmid DNA transfection was performed using PEI MAX (Polysciences) in ratio 1:6. HEK293 cells stably expressing pBIOID2-HA or pBIOID2-PPM1D-D314A have been described previously [49]. Silencer Select siRNA targeting TCOF1 (GCGAGGAAUCGGACAGUGAtt) or Negative Control (NC1) was transfected using RNAiMAX (both Thermo Scientific) at final concentration 5 nM, two subsequent rounds of siRNA transfection were performed, and cells were analyzed after 4 days. Cas9 expression in RPE-iCut cells was induced by overnight treatment with 2 μM doxycycline and 1 μM Shield-1 (Aobious), followed by transfection of sgRNA [52]. The formation of DSBs in rDNA was induced by transfection of 5 nM sgRNA (GCGGTGCGTGACGGGCGAGG) targeting 28S rDNA or sgRNA (TCCGAGATCAGACGAGATC) targeting 5S rDNA using Lipofectamine RNAiMAX (Thermo Fisher). RPE-HA-dTAG-PPM1D cells were generated by transfecting RPE cells with eSpCas9 (Sigma), sgRNA GUGAGCGUCUUCUCCGACCA targeting the exon 1 of PPM1D, and double-stranded DNA fragment coding for blasticidine resistance gene, P2A linker, HA-tag, and FKBP12^F36V^ sequence (described in [53]) flanked with 349–424 bp ling homology arms in 5′ UTR and exon 1 region of PPM1D gene. Cells were treated with blasticidine for 3 weeks, and single-cell clones were expanded. A positive clone expressing HA-dTAG-PPM1D was identified by immunoblotting, and correct targeting of one allele was validated by sequencing of the genomic DNA. The other allele contains a frameshift mutation in exon 1, preventing the expression of the non-tagged PPM1D. Where indicated, degradation of HA-dTAG-PPM1D was induced by incubation of cells with dTAGV-1 (TFA) (1 μM overnight, MedChemExpress, [54]).

### Antibodies and reagents

The following antibodies were used in this study with these dilutions: HA [sc-7392, 1:100 for IF, 1:50 for proximity ligation assay (PLA)], NPM1 (sc-271737, 1:100 for IF), POLR1A (sc-48385, 1:100 for IF, 1:50 for PLA), PPM1D (sc-20712, 1:100 for IF, 1:50 for PLA, sc-376257, 1:1000 for WB), and UBF (sc-13125, 1:1000) from Santa Cruz Biotechnology, PPM1D (#11901, 1:100 for IF), FBL (#2639, 1:300 for IF, 1:150 for PLA), pChk2-Thr68 (#2661, 1:1000 for WB), and phospho-CK2 motif (#8738, 1:500 for WB) from Cell Signaling Technology; pKAP1-Ser824 (NB100-2350, 1:1000 for WB) from Novus Biotechnologies, FLAG (ab1162, 1:100 for IF) from Abcam, EGFP (11 814 460 001, 1:200 for IF, 1:100 for PLA, and 1:1000 for WB) from Roche and, Tubulin (T5192, 1:1000 for WB) and TCOF1 (HPA038237, 1:300 for IF, 1:150 for PLA) from Sigma, and TCOF1 (11003–1-AP, 1:500 for WB) from Proteintech. PPM1D inhibitor GSK2830371 was from MedChemExpress and was validated previously [46, 55]. The CK2 inhibitor CX-4945 was obtained from MedChemExpress and used at final concentrations 1 μM [56]. Compounds CX-5461 and Actinomycin D (ActD) were obtained from MedChemExpress. Doxycycline for the induction of protein expression was used at 1 µM.

### Immunofluorescence microscopy

Cells grown on coverslips were washed in phosphate buffered saline (PBS), fixed by 4% formaldehyde for 15 min, and permeabilized with 0.2% Triton-X100 for 5 min. After washing in PBS, the coverslips were blocked with 1% bovine serum albumin (BSA) in PBS for 30 min, incubated with primary antibodies for 2 h at room temperature, and subsequently with Alexa Fluor secondary antibodies (Thermo Scientific) for 1 h. Where indicated, nucleolar dye TOTO-3 (Lumiprobe) was added. After incubation with DAPI for 2 min, coverslips were washed with water and mounted with Vectashield. For PLA, coverslips were stained with the indicated primary antibodies followed by incubation with PLA probes (Merck, Duolink *In Situ* PLA Probe Anti-Rabbit PLUS and MINUS, DUO92002, DUO92004) for 1 h at 37°C, ligation for 30 min at 37°C, and polymerase reaction for 100 min at 37°C according to the manufacturer’s protocol (Merck, Duolink *In Situ* Detection Reagents Red, DUO92008). For high-content microscopy, images were acquired using Olympus Evident ScanR equipped with 20×/0.8OIL or 40×/0.95 objectives, and analyzed using the ScanR analysis software. Neural networks for automated segmentation of nucleoli were custom-trained using CellSense software (Evident). Confocal imaging was performed using Leica DMi8 equipped with laser scanning confocal head (Leica STELLARIS 8) and an HC PL APO 63×/1.40 OIL CS2 objective. Images were acquired as Z-stacks of at least five planes with voxel size 52 × 52 × 139.7 nm, and 3D-deconvolved using Leica Lightning module.

### Fluorescence recovery after photobleaching

Fluorescence recovery after photobleaching (FRAP) data were acquired using inverted microscope (DMi8) with confocal head (Leica STELLARIS 8), equipped with an HC PL APO 63×/1.40 OIL CS2 objective and an incubation chamber to provide a humidified atmosphere at 37°C with 5% CO_2_. Cells were imaged in FluoroBrite DMEM (Gibco) medium supplemented with 10% FBS and 1× GlutaMAX (Gibco). Imaging was performed at a resolution of 200 × 200 pixels, a pixel size 84.3 × 84.3 nm, a scan speed of 600 Hz, and a pinhole set to 2.30 AU. The TCOF1-EGFP signal was bleached with 488-nm laser line selected by the AOTF from the WL laser with FRAP Booster beam expander. Imaging consisted of a pre-bleach sequence (3 × 0.34 s), followed by bleaching at 100% laser intensity (3 × 0.34 s), and a three-phase post-bleach monitoring period (40 × 1 s, 20 × 2 s, and 24 × 5 s) to capture the full recovery profile. Image processing was performed using Jython script in FIJI https://imagej.net/tutorials/analyze-frap-movies. The workflow included stack registration to correct for the cell movement, followed by intensity measurements of the bleached region. Data were corrected for incidental photobleaching using a control region, then background-subtracted, and normalized to accommodate a (0, 1) interval. Recovery curves were fitted to a one-phase exponential equation in GraphPad Prism. The immobile fraction of EGFP-TCOF1 was calculated as 1 – mobile fraction (where the mobile fraction represents the plateau of the fitted curve).

### TCOF1 partition and condensate size analysis

Cells were imaged using high-content ScanR microscope. Neural networks for automated segmentation of TCOF1 condensates were custom-trained using CellSense software (Evident). The free energy of transfer (ΔG^transfer^) was calculated based on K_partition_ as described [33, 57]. Dense and dilute phase concentrations were determined based on mean fluorescence intensity in and outside EGFP-TCOF1 condensates.

### Proximity biotinylation assay and mass spectrometry

The isolation of proteins interacting with PPM1D using HEK293 cells stably expressing pBIOID2 or pBIOID2-PPM1D-D314A and their identification by mass spectrometry have been previously reported [49], and the data are deposited in the PRIDE database (accession numbers PXD035268 and PXD035273).

### Phosphoproteomic analysis

Parental U2OS and U2OS-PPM1D-KO were grown in high-glucose DMEM supplemented with 6% FBS (Gibco), Penicillin (10 U/ml), and Streptomycin (0.1 mg/ml). Cells were treated with mock, 40 µM etoposide, or 1 µM PPM1D inhibitor (GSK2830371) for 2 h prior to harvest. The material was collected from two 15-cm dishes per condition in three biological replicates. Cells were lysed by boiling at 95°C for 10 min in 100 mM Tris, pH 8.5 containing 2% sodium deoxycholate, 40 mM chloroacetamide, and 10 mM Tris(2-carboxyethyl)phosphine, and were sonicated (Bandelin Sonoplus Mini 20, MS 1.5). Protein concentration was determined using BCA Protein Assay Kit (Thermo Scientific), and 250 µg of protein per sample was used for MS sample preparation. Proteins were digested by 5 µg of trypsin per sample at 37°C overnight. Phosphopeptides were enriched using TiO_2_ as described [58]. Nano reversed-phase columns (EASY-Spray column, 50 cm × 75 µm ID, PepMap C18, 2 µm particles, 100 Å pore size) were used for LC/MS analysis. Mobile phase buffer A was composed of water and 0.1% formic acid. Mobile phase B was composed of acetonitrile and 0.1% formic acid. Samples were loaded onto the trap column (C18 PepMap100, 5 μm particle size, 300 μm × 5 mm, Thermo Scientific) for 4 min at 18 μl/min. The loading buffer was composed of water, 2% acetonitrile, and 0.1% trifluoroacetic acid. Peptides were eluted with mobile phase B gradient from 2% to 35% B within 60 min. Eluting peptide cations were converted to gas-phase ions by electrospray ionization and analyzed on a Thermo Orbitrap Fusion (Q-OT-qIT, Thermo Scientific). Survey scans of peptide precursors from 350 to 1400 m/z were performed in orbitrap at 120K resolution (at 200 m/z) with a 1 × 10^6^ ion count target. Tandem MS was performed by isolation at 1.5 Th with the quadrupole, HCD fragmentation with normalized collision energy of 35, and rapid scan MS analysis in the ion trap. The MS2 ion count target was set to 10^4^, and the max injection time was 150 ms. Only those precursors with charge states 2–6 were sampled for MS2. The dynamic exclusion duration was set to 30 s with a 10 ppm tolerance around the selected precursor and its isotopes. All data were analyzed and quantified with the MaxQuant software (version 2.0.1.0) [59]. The false discovery rate was set to 1% for both proteins and peptides. The Andromeda search engine was used for the MS/MS spectra search against the human database from UniProt. Quantifications were performed with the label-free algorithm in MaxQuant [60]. Data analysis was performed using Perseus 1.6.15.0 software [61]. Only those phosphosites with a localization probability higher than 0.75 were used for further data analysis.

### Bioinformatic analysis

Gene Ontology (GO) enrichment analysis of phosphoproteomic data was done using ViSEAGO R package [62]. The sequence logo was plotted using ggseqlogo R package [63]. The kinase activities were predicted using the Fisher binary enrichment analysis available in the Kinase Library [64]. The multiple sequence alignment was performed using Clustal Omega [65]. The nucleolar localization sequence (NoLS) of PPM1D was predicted using the NoD server [66], and the conservation score was assessed using ConSurf server [67]. The dotplot visualization of TCOF1 LCRs was performed using EMBOSS Dotmatcher with threshold 10 and a window size 5 residues [65]. The structural prediction was performed using AlphaFold 3 server [68]. For each TCOF1 phosphopeptide, five different structures were obtained from a single seed, as is default for AlphaFold3. All seeds are listed in Supplementary Table S2.

### Immunoprecipitation

Expression of EGFP or EGFP-TCOF1-WT was induced in U2OS and U2OS-PPM1D-KO cells by overnight incubation with doxycycline. Cells were extracted with IP buffer (50 mM Tris, pH 8.0, 450 mM NaCl, 1% Tween 20, 0.1% NP-40, 10% glycerol, 2 mM ethylenediaminetetraacetic acid (EDTA), 3 mM ethylene glycol-bis(2-aminoethylether)-*N,N,N′,N′*-tetraacetic acid (EGTA), 1 mM Dithiothreitol (DTT), and 10 mM MgCl_2_) supplemented with PhosSTOP (Sigma) and cOmplete EDTA-free Protease Inhibitor Cocktail (Roche). After sonication for 3 × 20 s at 20% amplitude using Q125 Sonicator, samples were supplemented with 10 mM MgCl_2_ and 100 U/ml benzonase, and incubated for 30 min on ice. To ensure stripping of proteins from non-digested DNA, 50 μg/ml ethidium bromide was added, and the soluble fraction was obtained by centrifugation for 30 min at 4°C at 20 000 × *g*. After incubation with GFP Trap beads (Chromotek) at 4°C for 1 h, the beads were washed 5× with lysis buffer. Finally, immunoprecipitated proteins were eluted by incubation with sodium dodecyl sulfate (SDS) loading buffer for 5 min at 95°C, and analyzed using SDS–polyacrylamide gel electrophoresis (PAGE) and immunoblotting.

### *In vitro* kinase and phosphatase assay

The expression and purification of human His-PPM1D were described previously [69]. U2OS cell lines with inducible expression of EGFP-TCOF1-WT or EGFP-TCOF1-SA were incubated with doxycycline overnight. EGFP-TCOF1 was purified using GFP Trap and a high-salt IP buffer (50 mM Tris, pH 8.0, 450 mM NaCl, 1% Tween 20, 0.1% NP-40, 10% glycerol, 2 mM EDTA, 3 mM EGTA, and 10 mM MgCl_2_) supplemented with PhosSTOP and protease inhibitors. Beads were washed with a kinase buffer (10 mM Tris–HCL, pH 7.5, 2.5 mM DTT, 100 mM NaCl, 20 mM MgCl_2_, 0.15 mM ATP) and incubated with mock or with 200 ng of recombinant CK2 holoenzyme complex (PK-CK2ABH2-A010 from Biafinn) for 20 min at 30°C. Where indicated, beads were washed and then incubated with the purified 500 ng His-PPM1D in phosphatase buffer (50 mM HEPES, pH 7, 25 mM NaCl, 60 mM MgCl_2_, and 1 mM DTT) for 20 min at 37°C. The reaction was stopped by addition of 4× concentrated Laemmli buffer. Samples were analyzed using SDS–PAGE and immunoblotting.

### *In vitro* protein binding assay

Human EGFP-POLR1A was expressed in U2OS cells transiently transfected by p23-puro-EGFP-RPA194 (Addgene ID: 179237) and purified using a GFP Trap. A DNA fragment coding for amino acids 399–431 of human TCOF1 (containing LCR6 region, reported as fragment TCOF-F5 in [26]) was inserted in frame into BamHI/EcoRI sites of pGEX-6P1 plasmid, and the GST-fusion protein was purified from BL21 bacteria induced with 0.5 mM Isopropyl β-D-1-thiogalactopyranoside (IPTG). Recombinant GST or GST-TCOF1-LCR6 was incubated with CK2 in kinase buffer containing or not ATP for 20 min at 30°C. Proteins were then incubated with EGFP-POLR1A immobilized on beads for 1 h, and after extensive washing, bound proteins were analyzed by immunoblotting.

### 3D-TauSTED imaging

Cells grown on 1.5H precision coverslips (Marienfeld) were stained with primary antibodies against PPM1D and POLR1A, followed by incubation with secondary antibodies conjugated to STAR 580 or STAR 635P (Abberior). STED microscopy was carried out using a DMi8 microscope equipped with a STELLARIS 8 laser scanning confocal head. Five optical sections were acquired using a HC PL APO 100×/1.4 OIL STED WHITE CS2 oil immersion objective, with a pinhole size of 1 AU, in a 1504 × 1504-pixel format, and voxel dimensions of 22 × 22 × 130 nm. STAR 580 and STAR 635P dyes were excited sequentially using a white pulsed laser and depleted with a pulsed 775-nm STED laser. The tau-STED module (Leica), enabled by Fast Lifetime Contrast (FALCON), was used with HyD-X detectors and a time-gating window set between 0.3 and 6 nanoseconds. To reduce image noise, raw data were deconvolved using Xtend module (Leica).

### Structured illumination imaging

Three-dimensional structured illumination microscopy (3D-SIM) was conducted using a DeltaVision OMX™ V4 system equipped with the Blaze Module (GE Healthcare) and a PLAN APO N 60×/1.42 oil immersion objective. A 568-nm OPSL laser was used for excitation, and signal detection was carried out with a pco.edge 5.5 sCMOS camera. Raw images were acquired as z-stacks with 125-nm intervals across 8 z-slices, collecting 15 images per slice, with a pixel size of 80 nm. Image reconstruction was performed using SoftWorX software (GE Healthcare).

### Nascent RNA labeling

For 5-ethynyl-uridine (5EU) incorporation, cells were incubated with 1 mM 5EU (Lumiprobe) for 1 h at 37°C in 5% CO_2_. After incubation, cells were washed three times with PBS and fixed in 4% formaldehyde for 15 min. Incorporated 5EU was labeled via a Click-iT reaction carried out in a solution containing 0.1 M Tris (pH 8.5), 0.1 M sodium ascorbate, 2 mM CuSO_4_, and 10 μM AlexaFluor 568 azide (Lumiprobe) for 30 min at room temperature. Coverslips were then stained with the RNA stain TOTO-3 to enable quantification of 5EU signal specifically within the nucleoli. For cells expressing EGFP TCOF1 variants, only populations gated for comparable EGFP intensities were included in the analysis.

### Transmission electron microscopy

Cells were fixed in a solution of 1% glutaraldehyde and 4% formaldehyde (EM grade, Electron Microscopy Sciences) in PHEM buffer (60 mM PIPES, 25 mM HEPES, 10 mM EGTA, 2 mM MgCl_2_, pH 6.9) for 24 h at room temperature, followed by washing with 100 mM glycine to quench unreacted aldehydes. Cells were then gently scraped into 1% BSA in PHEM buffer and embedded in 1% low-melting-point agarose (Invitrogen). Post-fixation was performed using an aqueous mixture of 1% osmium tetroxide and 1.5% potassium ferricyanide for 4 h. Samples were subsequently stained with 1% uranyl acetate for 1 h, dehydrated through a graded ethanol series and 100% acetone, and embedded in Durcupan epoxy resin (Electron Microscopy Sciences). Ultrathin sections (75 nm) were prepared using a Leica EM UC7 ultramicrotome and imaged with a JEM-1400 FLASH transmission electron microscope (JEOL, Japan) equipped with a FLASH 2k × 2k CMOS camera (Hamamatsu Photonics, Japan), operating at 100 kV.

### Chromatin immunoprecipitation

Chromatin immunoprecipitation (ChIP) was performed using ChIP-IT Express kit following the manufacturer’s protocol (Active motif). Cells grown on 15-cm plate were fixed with 1% formaldehyde, and the reaction was quenched with 125 mM glycine for 5 min. Cells were then washed with PBS, scraped, and incubated in Lysis Buffer for 30 min on ice. Nuclei were isolated by Dounce homogenization and sonicated with 125 W sonicator 7 × 20 s at 25% amplitude. Cleared chromatin was incubated overnight with protein G magnetic beads and one of the following antibodies: TCOF1 (3 µg, Proteintech), RPA194 (2 µg, Santa Cruz), or GFP (2 µg, Millipore). Beads were washed three times, chromatin was eluted and decrosslinked for 2.5 h at 65°C, and digested with proteinase K for 1 h at 37°C. DNA was isolated with phenol-chloroform extraction and analyzed by quantitative polymerase chain reaction (qPCR) (LightCycler 480 II, Roche) using Luna Universal qPCR Master Mix (New England Biolabs) in 10-µl reaction volumes following the manufacturer’s recommended thermal cycling conditions. ChIP enrichment was calculated relative to the amount of input material based on cycle threshold (Ct) values and amplification efficiency: % ChIP = AE^(Input Ct - ChIP Ct)^ * (Fd) * 100 where: Fd = dilution factor of the input fraction, and AE = amplification efficiency calculated as 10^(−1/slope)^. The slope was determined from a standard curve using serial dilutions of the input template (0.005, 0.05, 0.5, 5, and 50 ng). Ct values were determined by LightCycler 480 Software using default settings, and mean Ct values were calculated from two technical replicates for each biological replicate. The melting curve analysis was performed to confirm single-product amplification. All primers and source data are described in Supplementary Table S3.

### Northern blotting

Total RNA was extracted from cells using RNeasy Mini Kit (Qiagen) and northern blotting was performed using the NorthernMax Kit (Invitrogen) according to the manufacturer’s instructions. Two to four micrograms of RNA was resolved on 1% formaldehyde-agarose gel for 2 h at 5 V/cm and blotted to a HYBOND-N + membrane (Cytiva) using capillary transfer. Membranes were exposed to 100 mJ/cm^2^ UV, dried, and prehybridized in ULTRAhyb Ultrasensitive Hybridization Buffer (Invitrogen) for 30 min at 37°C. Hybridization with ITS1 (CCTCGCCCTCCGGGCTCCGTTAATGATC) and ITS2 (GCGCGACGGCGGACGACACC-GCGGCGTC) biotinylated probes at final concentration 100 pM was carried out overnight at 37°C. The membrane was washed three times with Low Stringency Wash Solution #1 (Invitrogen), and hybridized probes were detected using Chemiluminescent Nucleic Acid Detection Module Kit (Thermo Scientific).

### Cell proliferation assay

Cells were plated in 96-well plates at a density of 100 (RPE) or 200 (MCF7) cells per well. Where indicated, cells were pre-treated with doxycycline, PPM1D inhibitor, or DMSO. After 24 h, cells were additionally treated with indicated doses of CX-5461 or ActD for 7 days. To assess cell viability, 30 μg/ml resazurin (Sigma, R7017) was added, and the cells were incubated for additional 3 h. Fluorescence was measured at 590 nm with excitation at 560 nm using an Envision plate reader (PerkinElmer). Measured values were normalized to the non-treated or PPM1Di-only-treated condition.

### Statistical analysis

Data visualization and statistical analysis were carried out using Prism 5 (GraphPad Software). Statistical significance in representative quantitative microscopy data was assessed using the two-tailed Mann–Whitney U test. Where appropriate, data with an assumed normal distribution were analyzed using an unpaired two-tailed *t*-test or two-way analysis of variance (ANOVA). In all graphs, data are presented as the mean, with error bars indicating the standard deviation. Statistical significance is denoted as follows: ****P < .0001, ***P < .001, **P < .01, *P < .05. All experiments were independently repeated at least twice with consistent results.

## Results

### PPM1D is targeted to the nucleolus by a nucleolar localization signal located at its C-terminus

We previously conducted a proximity-dependent proteomics screen to identify proteins that form complexes with PPM1D [49]. This approach revealed that PPM1D interacts with subunits of the shelterin complex and plays a role in maintaining telomere integrity [49]. In addition to shelterin components and other known PPM1D interactors, we identified several nucleolar proteins, including fibrillarin, NPM1, and nucleolin, suggesting a potential association of PPM1D with the nucleolus (Fig. 1A). To assess the physical association of PPM1D with the nucleolus, we performed confocal microscopy of U2OS and RPE cells, co-staining for nucleoli and PPM1D using the RNA-specific dye TOTO-3 and a previously validated PPM1D antibody (Fig. 1B). Consistent with the previous reports identifying PPM1D as a chromatin-bound protein, the majority of PPM1D was localized in the nucleus [35]. Nevertheless, a minor fraction of PPM1D was detected within the nucleolus. In contrast, PPM1G (another chromatin-associated phosphatase from the PP2C family [70]) was clearly excluded from the nucleolus (Supplementary Fig. S1A). Nucleolar localization is commonly mediated by short basic motifs known as NoLSs [71]. Interestingly, the NoLS detector software (NoD), predicted a NoLS within the 572–596 region of PPM1D (Fig. 1C) [66]. Although the disordered C-terminus of PPM1D is generally less evolutionary conserved than the catalytic domain, the basic residues spanning positions 581–589 within the predicted region are conserved across species (Fig. 1C and Supplementary Fig. S1B). To validate the predicted NoLS, we expressed EGFP-tagged PPM1D or its deletion mutants in U2OS-PPM1D-KO cells lacking the endogenous PPM1D. The catalytic domain (amino acids 1–380) failed to localize to the nucleolus, as did a PPM1D-ΔNoLS mutant lacking the conserved basic residues 581–589 (Fig. 1D–F). In contrast, the unstructured C-terminal fragment of PPM1D (residues 375–605) showed clear enrichment in the nucleolus (Fig. 1E and F). To prove the ability of endogenous PPM1D to localize to the nucleoli, we performed PLA using established nucleolar markers. Indeed, we observed a PLA signal between PPM1D, fibrillarin, and UBF in U2OS cells, whereas the signal was lost in U2OS-PPM1D-KO cells lacking PPM1D (Fig. 1G). Next, we evaluated the localization of PPM1D using 3D-STED and 3D-SIM, and found colocalization of PPM1D with RNA Pol I, indicating that PPM1D can localize to the FCs where the rDNA transcription occurs (Fig. 1H and Supplementary Fig. S1C). Finally, we used confocal microscopy to quantify the colocalization of PPM1D with FCs stained for RNA Pol I and UBF (Fig. 1I). This analysis revealed that PPM1D was present in ~9% of FCs. Taken together, these results confirm that a fraction of PPM1D localizes to the nucleolus via a NoLS located within its C-terminus, and that PPM1D colocalizes with several DFC and FC factors, including RNA Pol I, which is responsible for rDNA transcription.

**Figure 1.**
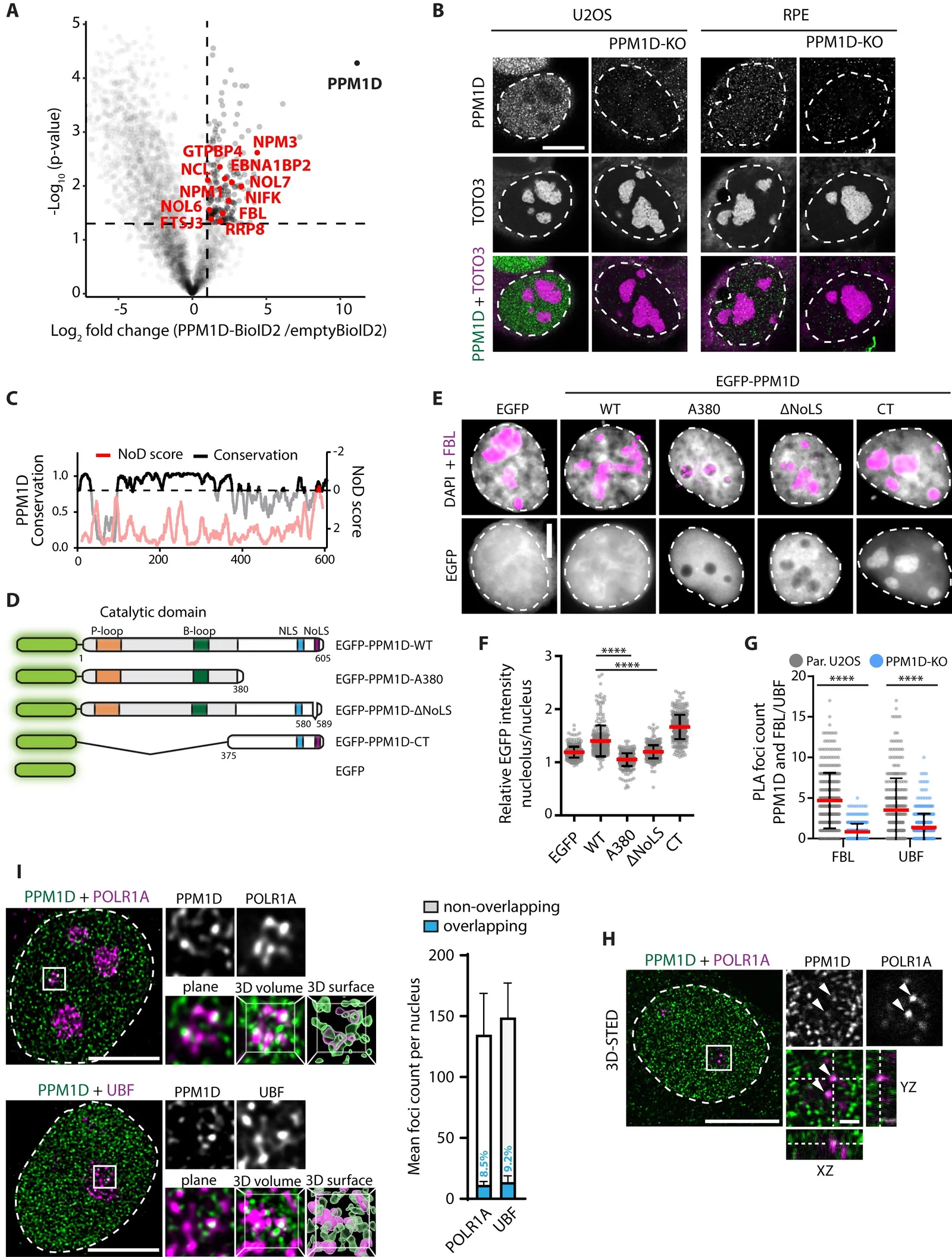
PPM1D is targeted to the nucleolus by nucleolar localization signal. (**A**) HEK293 cells stably expressing either PPM1D-D314A-BioID2 or the empty BioID2 vector were lysed 5 h after biotin treatment. Biotinylated proteins were enriched using streptavidin beads and analyzed by mass spectrometry (*n* = 3). The volcano plot displays –log_10_  *P*-values for proteins enriched or depleted in the PPM1D-BioID2 sample relative to control. The dashed lines indicate the threshold for statistical significance (*P* < .05) and a log_2_ fold change > 1. Proteins with nucleolar localization and function are highlighted in red. (**B**) RPE and U2OS parental or PPM1D-KO cells were stained with TOTO3 and an anti-PPM1D antibody and were imaged by confocal microscopy. Representative single deconvolved planes are shown. The scale bar represents 10 µm. (**C**) Conservation score assessed for the protein sequence of PPM1D using ConSurf, plotted as the moving average of 5-residue window (in black). On the right *y*-axis, the score from Nucleolar detector is shown, corresponding to moving 20-residue window. Values above the dashed line are considered as conserved (<0) or candidate NoLS (>0.8), respectively. (**D**) Schematic representation of EGFP-PPM1D mutants used in the study. (**E**) High-content microscopy analysis of the localization of PPM1D deletion mutants. U2OS cells were transiently transfected with plasmids coding for EGFP-PPM1D constructs. Cells were fixed and stained for NCL to allow segmentation of nucleoli. Representative images are shown, the scale bar represents 10 µm. (**F**) Quantification of E. The EGFP intensity nucleolus/nucleus ratio from representative experiment is shown. The mean ± SD is plotted (*n* ≥ 385), Mann–Whitney test (****P < .0001). A representative experiment is shown from two independent repeats. (**G**) Parental and PPM1D-KO U2OS cells were fixed and probed for interaction of PPM1D with Fibrillarin or UBF by PLA assay. The mean ± SD is shown from representative experiment (*n* ≥ 327), Mann–Whitney test (****P < .0001). (**H**) 3D-STED imaging of MCF7 cells stained with PPM1D and POLR1A antibody (scale bars 10 and 1 µm). Shown are a deconvolved single plane and an enlarged nucleolar region with XZ and YZ orthogonal sections indicated by dashed lines. (**I**) Representative confocal images of cells stained with PPM1D and POLR1A (top) or UBF (bottom). The scale bar indicates 10 µm; insets show deconvolved single optical sections, along with corresponding 3D volume and surface renderings. Quantification of 3D surfaces using Imaris software showing number of POLR1A or UBF foci that overlap with ≥50% of its volume with PPM1D surfaces. The mean ± SD is shown from three independent experiments (*n* = 3). Numbers indicate the fraction of colocalizing foci.

### PPM1D is not essential for initiation of the nucleolar DNA damage response

PPM1D is known to contribute to termination of the global DNA damage response by dephosphorylating substrates of ATM and ATR kinases [37, 47, 72]. In addition, PPM1D has been shown to promote the repair of double-strand breaks (DSBs) through homologous recombination [51]. Therefore, we asked whether PPM1D plays a role in the repair of rDNA breaks, which are repaired via a specific mechanism involving alternative ATM activation and subsequent relocalization of rDNA repeats to the nucleolar periphery preventing ectopic recombination [25, 73]. To induce breaks specifically in rDNA, we utilized RPE-iCut cells with inducible Cas9 expression and transfected them with sgRNA targeting repeats coding for 28S rRNA [74]. As expected, the induction of rDNA breaks led to relocalization of UBF to the nucleolar caps (Supplementary Fig. S2A). Formation of the nucleolar caps remained unchanged upon PPM1D inhibition, suggesting that PPM1D is not required for activation of nucleolar DDR. Next, we asked whether PPM1D inhibitor affects levels of γH2AX, which is a well-known substrate of PPM1D [35, 75]. As expected, cells treated with PPM1D inhibitor showed elevated γH2AX levels in the nucleolar caps (Supplementary Fig. S2B). Recent findings show that γH2AX is required for efficient localization of the recombinase RAD51 in nucleolar caps and for repair of the nucleolar breaks [76]. We found that PPM1D inhibition did not affect RAD51 recruitment to nucleolar caps, suggesting that HR-mediated repair of the nucleolar DNA breaks is likely not affected (Supplementary Fig. S2C). To test this on the level of cell viability, we treated RPE1-iCut with low concentrations of sgRNA targeting the nucleolar DNA. Concomitant treatment with PPM1D inhibition did not further sensitize cells to breaks in 28S rDNA, and no effect was found also when DNA breaks were induced in 5S rRNA genes that are located outside nucleolus (Supplementary Fig. S2D). These results indicate that PPM1D does not control the initial nucleolar DNA damage response, although we have not formally excluded its involvement in completion or fidelity of nucleolar DSB repair.

### PPM1D dephosphorylates TCOF1 at low-complexity repeats

As a negative regulator of the DNA damage response, PPM1D is known to dephosphorylate SQ/TQ phosphorylation sites (commonly targeted by ATM, ATR, and DNA-PK kinases), with a preference for motifs enriched in glutamic acid and located within IDRs [47, 77]. Using a phosphoproteomic screen, we compared the effect of a DSB-inducing agent etoposide in control U2OS cells, U2OS-PPM1D-KO cells lacking PPM1D, and U2OS cells treated with PPM1D inhibitor, and fully confirmed these findings (Fig. 2A–C, Supplementary Fig. S3A and B, and Supplementary Table S1). In addition, we analyzed the differences in basal phosphorylation (in the absence of exogenous DNA damage) and found that 739 phosphorylations were upregulated in U2OS-PPM1D-KO cells and in U2OS cells treated with PPM1D inhibitor compared to the parental nontreated U2OS cells, suggesting that PPM1D regulates a subset of phosphorylation sites that are not induced by DNA damage (Fig. 2D). These phosphorylation sites were generally enriched in glutamic acid, aspartic acid, and serine residues and differed from the SQ/TQ consensus observed after DNA damage (Fig. 2D and Supplementary Fig. S3C). Notably, the GO Term Enrichment analysis revealed that these phosphorylation events occurred also in proteins involved in ribonucleoprotein biogenesis (Fig. 2E and Supplementary Fig. S3D). Among these, we noted that phosphorylation of several residues in TCOF1 responded to PPM1D activity and scored among the top hits consistently in PPM1D-KO cells or cells treated with PPM1D inhibitor (Fig. 2F). The consensus motif preference of PPM1D overlaps with the S/E-rich LCRs of TCOF1, which were previously reported to be phosphorylated by CK2 [23, 24, 78]. Moreover, the kinase prediction of the identified phosphopeptides suggested that PPM1D can dephosphorylate the CK2 substrates (Supplementary Fig. S3E). To test the regulation of TCOF1 modification by PPM1D, we immunoprecipitated EGFP-TCOF1 from U2OS cells and phosphorylated it with recombinant CK2 *in vitro*. Subsequent incubation with the catalytic domain of PPM1D led to a marked reduction in the phosphorylation, as detected by a phospho-CK2 motif-specific antibody (Fig. 2G and H). In contrast, the EGFP-TCOF1-SA mutant, in which all CK2 target serine residues across all LCRs were mutated to alanine, showed no detectable phosphorylation (Fig. 2G and H; Supplementary Fig. S3F).

**Figure 2.**
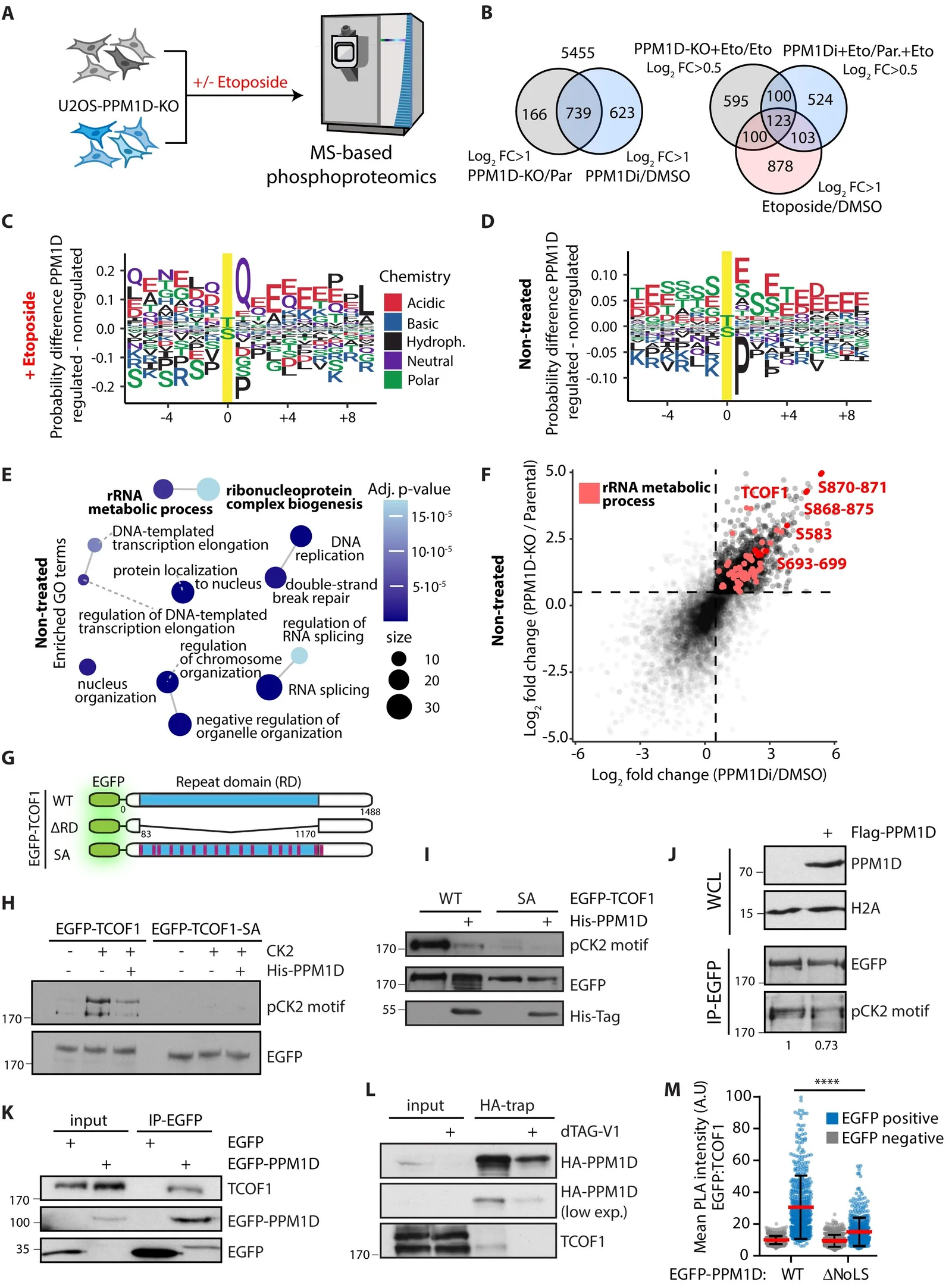
PPM1D dephosphorylates TCOF1 at low-complexity repeats. (**A**) Schematic representation of the phosphoproteomic screen. (**B**) Venn diagram representing overlap of phosphorylations enriched in PPM1D-KO cells and PPM1Di treated cells in unperturbed conditions (left). The overlap of phosphorylations detected in etoposide-treated cells is shown in the right. (**C**) Sequence motif analysis of PPM1D-regulated phosphorylations induced by etoposide. Amino acid probability difference is shown between 123 PPM1D-regulated and etoposide-induced phosphorylations and 878 PPM1D-nonregulated and etoposide-induced phosphorylations. (**D**). Amino acid probability difference is shown between 739 PPM1D-regulated phosphorylations and 5455 PPM1D-nonregulated phosphorylations. (**E**) GO Biological Process term analysis of PPM1D-regulated phosphorylations using ViseaGO R package. The 12 most enriched terms are shown, *P*-values were calculated using Fisher exact test. (**F**) Dot plot showing Log_2_ fold change of phosphorylations in PPM1D-KO cells and PPM1Di-treated cells relative to controls. The dashed lines indicate threshold log_2_ FC > 1. Enriched hits from rRNA metabolic process GO term are shown in red. TCOF1 phosphopeptides are highlighted. (**G**) Schematic diagram of the wild-type EGFP-TCOF1 and its mutants used in the study. (**H**) Immunoprecipitated EGFP-TCOF1 WT and the non-phosphorylable SA variant were incubated with recombinant CK2 kinase in presence of ATP for 30 min. Upon washing, recombinant His-PPM1D was added for 30 min where indicated. The phosphorylation was detected using immunoblotting with phospho-CK2-motif antibody. (**I**) EGFP-TCOF1 and EGFP-TCOF1-SA mutant were purified from cells using GFP trap, and the beads were incubated or not with purified His-PPM1D. Phosphorylation was determined by immunoblotting with a phospho-CK2-motif antibody. (**J**) Expression of EGFP-TCOF1 in U2OS-PPM1D-KO cells and U2OS-PPM1D-KO cells reconstituted with FLAG-PPM1D was induced with overnight doxycycline treatment. Cell extracts were incubated with GFP Trap. Phosphorylation of EGFP-TCOF1 was detected using immunoblotting with phospho-CK2-motif antibody. Numbers indicate phospho-CK2 motif intensity relative to U2OS-PPM1D-KO cells, normalized to total immunoprecipitated EGFP-TCOF1. (**K**) Expression of EGFP and EGFP-PPM1D in U2OS was induced with overnight doxycycline treatment. Cell extracts supplemented with benzonase were incubated with GFP Trap. Binding of TCOF1 was probed by immunoblotting. (**L**) Cell extracts from RPE-HA-dTAG-PPM1D cells treated with DMSO or dTAGV-1 (1 µM, overnight) were incubated with HA-trap beads, washed, and bound proteins were analyzed by immunoblotting. (**M**) Expression of EGFP-PPM1D-WT and the ΔNoLS variant was induced with overnight doxycycline treatment. Cells were fixed and probed for the interaction of EGFP-PPM1D with TCOF1 by PLA assay. The mean PLA intensity ± SD is shown from representative experiment (*n* = 500); Mann–Whitney test (****P < .0001).

To determine the phosphorylation of TCOF1 *in vivo*, we immunopurified EGFP-TCOF1 from the control or CK2 inhibitor-treated cells. We noted that inhibition of CK2 increased the electrophoretic mobility of EGFP-TCOF1 and it also abolished its recognition with the antibody directed to the phosphorylated CK2 motifs (Supplementary Fig. S3G and H). Further, we found that while the wild-type TCOF1 was strongly positive for the phosphorylated CK2 motifs, the signal was reduced after *in vitro* dephosphorylation by PPM1D and in TCOF1-SA mutant confirming that TCOF1 was phosphorylated at LCRs in cells (Fig. 2I). Next, we immunoprecipitated EGFP-TCOF1 from the PPM1D-KO cells and PPM1D-KO cells reconstituted with the catalytically active PPM1D (Fig. 2J). We found that PPM1D expression significantly reduced the level of TCOF1 phosphorylation at CK2 motifs, supporting the conclusion that PPM1D can dephosphorylate TCOF1 in cells (Fig. 2J).

Finally, we aimed to address how PPM1D localization can influence its effect on TCOF1. To this end, we first confirmed the interaction of EGFP-PPM1D with endogenous TCOF1 using co-immunoprecipitation from the transfected U2OS cells (Fig. 2K). Similarly, HA-dTAG-tagged PPM1D expressed from the endogenous promoter in RPE cells showed interaction with TCOF1, and, as expected, this binding was lost after induction of PPM1D degradation by incubation of cells with dTAGV-1 compound (Fig. 2L, Supplementary Figs S3I and 4A). The majority of PPM1D is localized at chromatin and relatively small fraction is present in nucleoli. Inversely, TCOF1 primarily localizes to the nucleolus, but it has also been observed at chromatin, including telomeres [79, 80]. Therefore, we performed PLA to characterize the colocalization of PPM1D and TCOF1 at subcellular level. We found that EGFP-PPM1D-ΔNoLS mutant showed reduced proximity to the endogenous TCOF1 compared to the wild-type EGFP-PPM1D, suggesting that nucleolar localization is important for this interaction (Fig. 2M and Supplementary Fig. S4B). Collectively, the results from an unbiased proteomic screen and *in vitro* biochemical assays indicate that PPM1D can directly dephosphorylate CK2-mediated phosphorylation of TCOF1 LCRs.

### Phosphorylation promotes TCOF1 condensation and regulates the mobility of TCOF1 in fibrillar centers

Recent studies have shown that the S/E-rich LCRs of TCOF1 promote its condensation in the nucleolus, likely through electrostatic interactions between negatively charged S/E-rich stretches and positively charged regions within the RD [33, 34]. Given that phosphorylation alters the charge distribution within RD, we investigated its role in TCOF1 condensation. To this end, we quantified the nucleolar localization of EGFP-tagged TCOF1 variants (Fig. 2G). As expected, the wild-type EGFP-TCOF1 displayed strong nucleolar enrichment, consistent with a single-component phase behavior (Fig. 3A and Supplementary Fig. S4C). In contrast, the TCOF1-ΔRD variant, lacking the RD (residues 83–1170), was present in the nucleoli, but it also localized to the soluble nucleoplasmic fraction, suggesting impaired condensation (Fig. 3A and Supplementary Fig. S4C). Similarly, the TCOF1-SA variant showed significantly lower nucleolar enrichment compared to the wild-type TCOF1 thus failing to display the expected single-component condensation (Fig. 3A and Supplementary Fig. S4C). Notably, the inhibition of CK2 led to impaired nucleolar localization of the expressed wild-type EGFP-TCOF1 (Fig. 3B and Supplementary Fig. S4D). A similar defect in EGFP-TCOF1 localization was observed upon PPM1D overexpression, which is expected to promote TCOF1 dephosphorylation (Fig. 3B and Supplementary Fig. S4D). To further investigate TCOF1 self-assembly, we employed a previously established biophysical approach to quantify the free energy of transfer (ΔG^transfer^), which reflects the favorability of a protein to partition into the dense phase [33, 57]. ΔG^transfer^ was calculated based on the relative concentrations of TCOF1 in the dense (condensate) and dilute (surrounding) phases. For TCOF1 WT, ΔG^transfer^ remained constant across varying expression levels, consistent with concentration-dependent expansion of the dense phase—indicative of self-assembly independent of other components (Fig. 3C). In contrast, both the ΔRD and SA variants exhibited saturation of the dense phase at higher expression levels, as shown by an increase in ΔG^transfer^ and only minimal growth in condensate size (Fig. 3C). This behavior suggests that these variants have a reduced ability to self-assemble. Because TCOF1 condensation plays a critical role in establishing the structure of the FC within the nucleolus, we next examined the ability of TCOF1 variants to exclude the GC marker NPM1. Unlike TCOF1 WT, the ΔRD and SA variants failed to exclude NPM1 and instead mixed with it, confirming their impaired ability to self-assemble (Fig. 3D). Finally, we tested whether the non-phosphorylatable SA variant could engage in homotypic interactions with TCOF1-WT. While the HA-TCOF1-WT and EGFP-TCOF1-WT were mixed within the condensates, the EGFP-TCOF1-SA mutant was excluded from the HA-TCOF1-WT condensates, further demonstrating a defect in self-association (Fig. 3E). Together, these findings suggest that phosphorylation, rather than intrinsic charge alone, is essential for proper nucleolar condensation of TCOF1.

**Figure 3.**
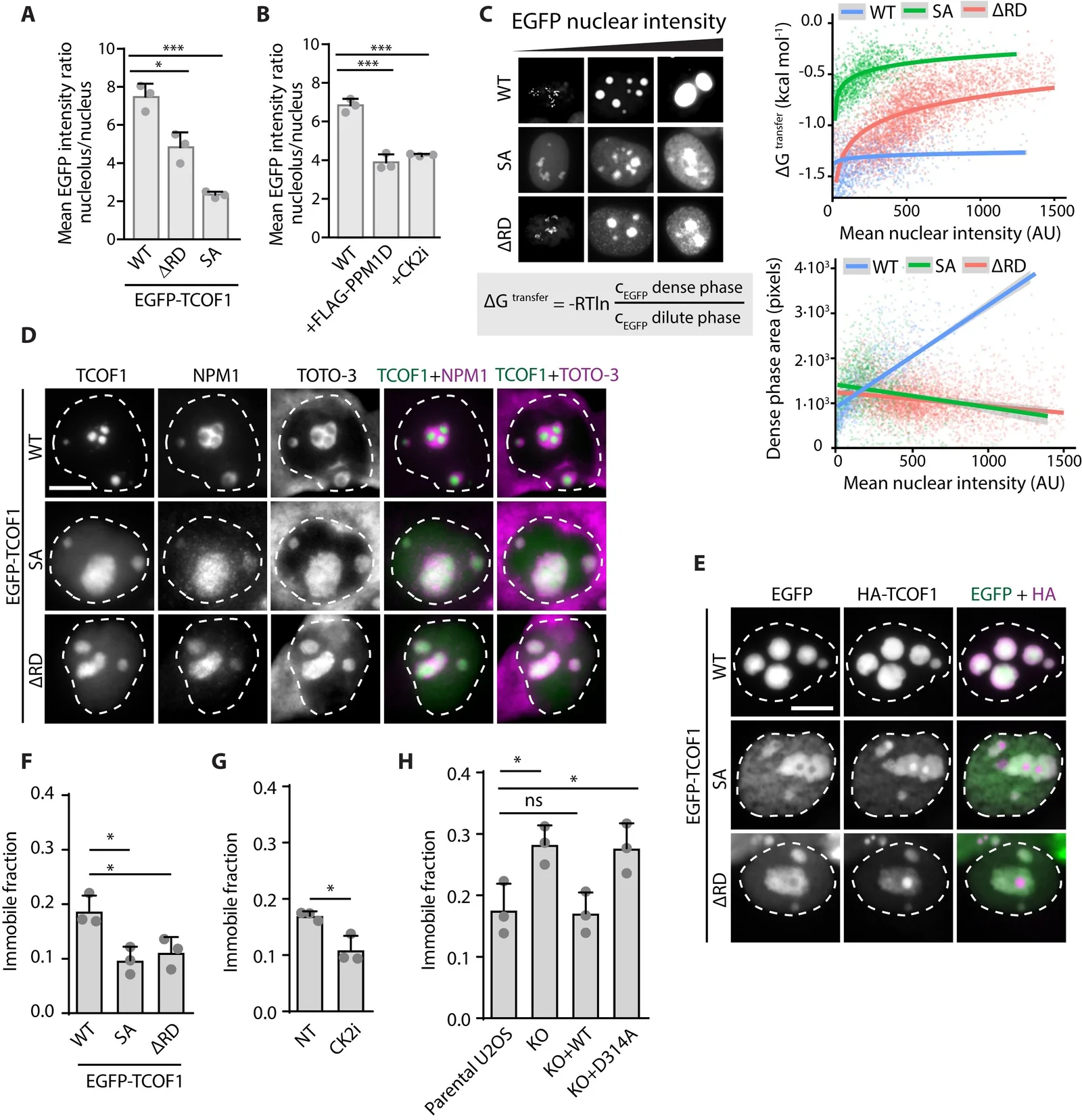
Phosphorylation promotes TCOF1 condensation and regulates mobility of TCOF1 in FC. (**A**) Expression of the wild-type EGFP-TCOF1, SA, and ΔRD variants was induced by overnight doxycycline treatment. The ratio between mean nucleolar and nuclear intensity is shown from three independent experiments. Nucleoli were segmented based on TOTO-3 staining. The error bars indicate SD, Statistical significance was evaluated using two-tailed *t*-test (*P < .05, ***P < .001). (**B**) Expression of the EGFP-TCOF1-WT was induced for 6 h with doxycycline. Where indicated, cells were transfected with plasmid coding for FLAG-PPM1D or treated with CK2i 3 h prior fixation. Cells were then stained with TOTO-3 and anti-FLAG antibody. The ratio between mean nucleolar and nuclear intensity is shown from three independent experiments. Nucleoli were segmented based on TOTO-3 staining. The error bars indicate SD, statistical significance was evaluated using two-tailed *t*-test (***P < .001). (**C**) Relation of ΔG^transfer^ and mean nuclear intensity in U2OS cells expressing the wild-type EGFP-TCOF1, SA, and ΔRD variants upon induction with doxycycline for 48 h. Logarithmic fits and 95% confidence intervals are shown (*n* > 1200). Relation of total dense phase area and mean nuclear intensity in U2OS cells expressing the wild-type EGFP-TCOF1, SA, and ΔRD variants upon induction with doxycycline for 48 h. Linear fits and 95% confidence intervals are shown (*n* > 1200). Representative images of cells expressing low, medium, and high levels of EGFP are shown on the left. For better visualization, the image intensities were independently scaled for each EGFP expression level. The formula for calculation of energy of partitioning (ΔG^transfer^) is shown below. R is the gas constant, T is the temperature, and c_EGFP_ correspond to concentration of EGFP measured as mean fluorescence intensity in either dense or dilute phase. (**D**) Expression of EGFP-TCOF1, SA, and ΔRD variant in U2OS cells was induced overnight with doxycycline. Cells were then stained with TOTO-3 and an anti-NPM1 antibody. The scale bar represents 10 µm. (**E**) Cells were transfected with plasmid coding for HA-TCOF1-WT 24 h prior induction of EGFP-TCOF1, SA, and ΔRD variant expression with overnight doxycycline treatment. Cells were then fixed and stained for HA. The scale bar represents 10 µm. (**F**) Immobile fraction of wild-type EGFP-TCOF1, SA, and ΔRD variants in U2OS cells was analyzed by FRAP. The EGFP-TCOF1 expression was induced by overnight doxycycline treatment. The mean is shown ± SD from three experiments, *n* ≥ 8, two-tailed *t*-test (*P < .05). (**G**) Expression of the EGFP-TCOF1-WT in U2OS cells was induced for 6 h with doxycycline. Where indicated, cells were treated with CK2i 3 h prior fixation. The mean percentage of the immobile fraction is shown ± SD from three experiments, *n* ≥ 8, two-tailed *t*-test (*P < .05). (**H**) Parental U2OS, U2OS-PPM1D-KO, and U2OS-PPM1D-KO cells stably expressing FLAG-PPM1D WT or D314A mutant were treated overnight with doxycycline to induce EGFP-TCOF1-WT expression. Immobile fraction was analyzed by FRAP. The mean is shown ± SD from three experiments, *n* ≥ 9, two-tailed *t*-test (*P < .05, ns *P* ≥ .05).

The RD domain has previously been implicated in physical association of TCOF1 with the rDNA [20]. To determine whether phosphorylation regulates the immobile fraction of TCOF1 within the FC, we performed FRAP on cells expressing different EGFP-TCOF1 variants. To ensure physiological relevance, we selected cells with low expression levels of EGFP-TCOF1 that maintain the normal GC structure. As expected, both EGFP-TCOF1-ΔRD and EGFP-TCOF1-SA mutants exhibited a significantly reduced immobile fraction compared to the wild-type TCOF1 (Fig. 3F and Supplementary Fig. S4E). Similarly, treatment with a CK2 inhibitor decreased the immobile fraction of EGFP-TCOF1-WT, indicating that phosphorylation contributes to TCOF1 stabilization within the FC (Fig. 3G and Supplementary Fig. S4F). To further assess the role of PPM1D in this regulation, we analyzed EGFP-TCOF1 mobility in parental U2OS cells, PPM1D-KO cells, and PPM1D-reconstituted cells. Expression of the catalytically active PPM1D (but not phosphatase dead D314A mutant) significantly reduced the immobile fraction of TCOF1, consistent with the impact of PPM1D-mediated dephosphorylation (Fig. 3H and Supplementary Fig. S4G). Together, these results demonstrate that the phosphorylation status of TCOF1 dictates its immobile fraction in the FC.

### Reversible phosphorylation of TCOF1 promotes its interaction with RNA polymerase I

TCOF1 has been reported to interact through its RD with POLR1A, the catalytic subunit of Pol I, but the role of phosphorylation in this interaction has not been addressed [19]. To this end, we performed PLA and found that the PLA signal between POLR1A and EGFP-TCOF1-SA was significantly reduced compared to the EGFP-TCOF1-WT (Fig. 4A). Similarly, EGFP-TCOF1-ΔRD showed a marked decrease in its association with POLR1A (Fig. 4A). As expected, we also observed co-immunoprecipitation of POLR1A with the wild-type TCOF1, and this was reduced in the TCOF1-SA mutant (Supplementary Fig. S5A). These data suggest that the binding of POLR1A depends on phosphorylation of the RD domain of TCOF1, thus resembling a previously reported interaction between TCOF1 and CUL3^KBTBD8^ [26, 27]. Moreover, CK2 inhibition led to diminished PLA signal for the TCOF1-WT:POLR1A interaction, but not for the SA variant, confirming that CK2-mediated phosphorylation enhances this interaction (Fig. 4A). In addition, EGFP-TCOF1-SA and EGFP-TCOF1-ΔRD were deficient in colocalization with UBF, suggesting that phosphorylation of LCRs may be involved in mediating TCOF1 interactions with multiple nucleolar proteins (Supplementary Fig. S5B). Functionally, expression of the EGFP-TCOF1-SA in cells depleted for endogenous TCOF1 led to reduced incorporation of 5EU compared to expression of the wild-type EGFP-TCOF1, suggesting that phosphorylation of TCOF1 is needed for rDNA transcription (Fig. 4B and Supplementary Fig. S5C and D). Similarly, EGFP-TCOF1-ΔRD showed a decreased rDNA transcription (Fig. 4B and Supplementary Fig. S5C and D).

**Figure 4.**
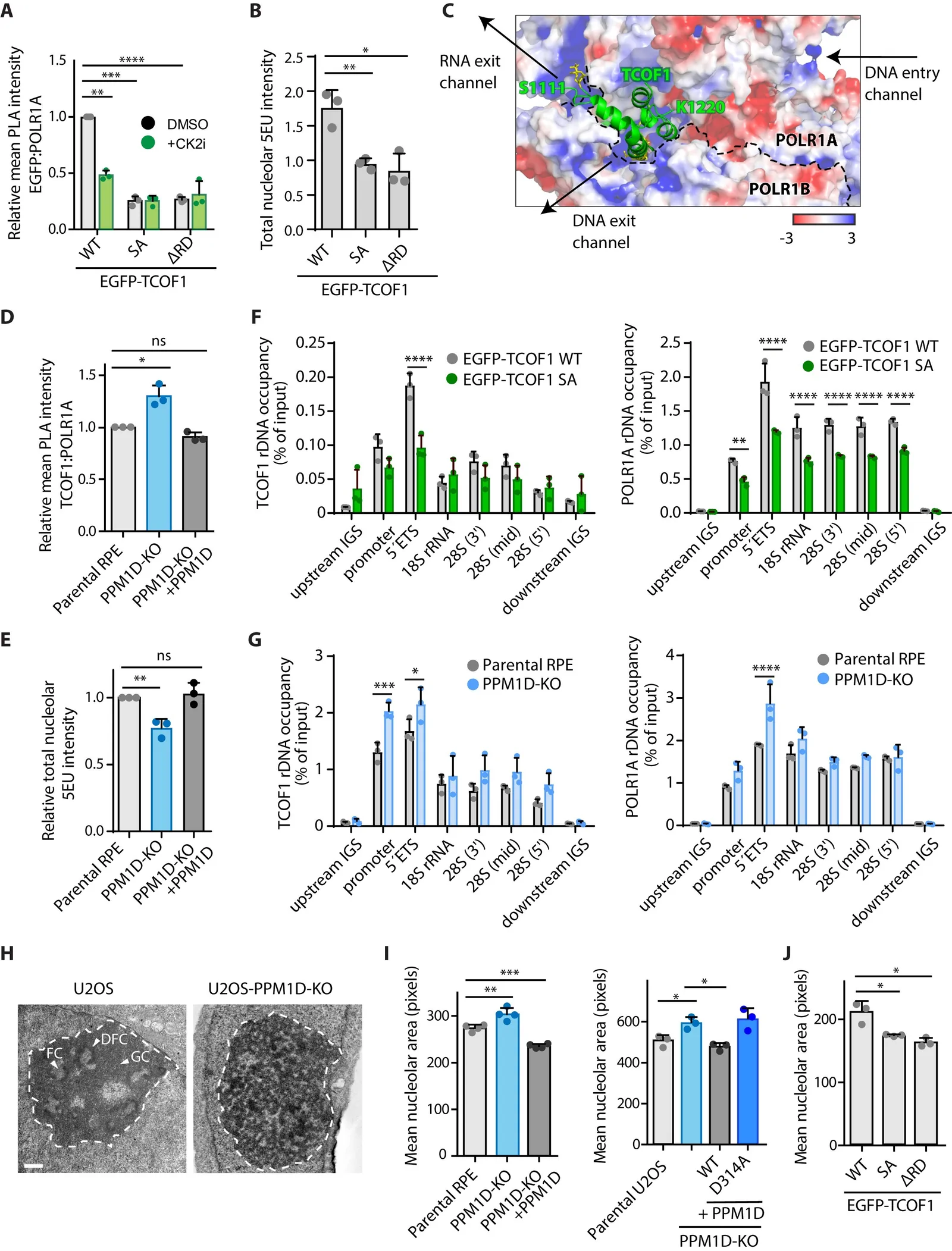
Reversible phosphorylation of TCOF1 promotes its interaction with RNA Pol I. (**A**) Expression of wild-type EGFP-TCOF1, SA, and ΔRD variants in U2OS was induced for 6 h with doxycycline. Where indicated, cells were treated with CK2i 2 h prior fixation. Cells were then probed for interaction between EGFP and POLR1A using PLA. The relative mean intensity ± SD is shown from three independent experiments; statistical significance was evaluated using two-tailed *t*-test (**P < .01, ***P < .001, ****P < .0001). (**B**) Cells were depleted for endogenous TCOF1 for 4 days by repeated transfection of siRNA. Expression of siRNA-resistant wild-type EGFP-TCOF1, SA, and ΔRD variants were induced by overnight treatment with doxycycline. Nascent RNA was pulse-labeled by 5EU treatment for 1 h. Cells were fixed, and incorporated 5EU labeled by click-IT chemistry. Nucleoli were segmented based on TOTO-3 staining. The total nucleolar 5EU intensity relative to EGFP-negative cells ± SD is shown from three independent experiments; statistical significance was evaluated using two-tailed *t*-test (*P < .05, **P < .01). (**C**) Interaction of TCOF1 (with phosphorylated serines in repeats 16 and 17) with POLR1A and POLR1B predicted by AlphaFold 3 server. TCOF1 is shown in green, with only residues 1111–1220 displayed for clarity. Phosphorylated serine residues are highlighted in yellow. Representation of POLR1A and POLR1B surface is colored by electrostatic potential that was determined using APBS Electrostatics Plugin in PyMOL (positive potential in blue, negative potential in red). (**D**) Parental RPE, PPM1D-KO, and PPM1D-KO cells expressing EGFP-PPM1D WT were fixed and probed for interaction between TCOF1 and POLR1A by PLA. The relative mean intensity ± SD is shown from three independent experiments; statistical significance was evaluated using two-tailed *t*-test (*P < .05, ns *P* ≥ .05). (**E**) Parental RPE, PPM1D-KO, and PPM1D-KO cells expressing EGFP-PPM1D WT treated with 5EU for 1 h. Nascent RNA was then labeled by click-IT chemistry. Nucleoli were segmented based on TOTO-3 staining. The relative mean nucleolar intensity ± SD is shown from three independent experiments; statistical significance was evaluated using two-tailed *t*-test (**P < .01, ns *P* ≥ .05). (**F**) Occupancy of EGFP-TCOF1 and POLR1A at various regions of rDNA gene was determined in cells expressing the wild-type EGFP-TCOF1 or EGFP-TCOF1-SA mutant using ChIP-qPCR. Mean value from three experiments ± SD normalized to the % of input is plotted. Statistical significance was determined by two-way ANOVA with Šídák’s multiple comparisons test (**P < .01, ****P < .0001). (**G**) Occupancy of TCOF1 and POLR1A at various regions of rDNA gene was determined in parental RPE and RPE-PPM1D-KO cells using ChIP-qPCR. Mean value from three experiments ± SD normalized to the % of input is plotted. Statistical significance was determined by two-way ANOVA with Šídák’s multiple comparisons test (*P < .05, **P < .01, ****P < .0001). (**H**) Parental U2OS and PPM1D-KO cells were imaged by transmission electron microscopy. The scale bar corresponds to 1 µm. Representative images are shown. (**I**) Parental RPE, PPM1D-KO, and PPM1D-KO cells expressing EGFP-PPM1D WT were fixed and stained with TOTO-3. Parental U2OS, U2OS-PPM1D-KO, and U2OS-PPM1D-KO cells stably expressing FLAG-PPM1D WT or D314A mutant were fixed and stained for fibrillarin. Mean nucleolar area ± SD is shown from four independent experiments; statistical significance was evaluated using two-tailed *t*-test (*P < .05, **P < .01, ***P < .001). (**J**) Cells were depleted for endogenous TCOF1 for 4 days by repeated transfection of siRNA. Expression of siRNA-resistant wild-type EGFP-TCOF1, SA, and ΔRD variants were induced by overnight treatment with doxycycline. Nucleoli were segmented based on TOTO-3 staining. Mean nucleolar area ± SD is shown from three independent experiments; statistical significance was evaluated using two-tailed *t*-test (*P < .05).

To gain structural insight, we used AlphaFold to model the interaction between POLR1A and phosphorylated TCOF1. The models revealed that phosphorylation of CK2 motifs within the RD reduced the predicted alignment error, indicating a more confident interaction (Fig. 4C and Supplementary Fig. S5E). The predictions indicated that the motifs in the RD region phosphorylated by CK2 bind to the positively charged DNA-binding cleft of POLR1A, with no apparent preference for the specific repeats (Supplementary Fig. S5F). This mechanism would require reversible phosphorylation, similar to the reported CK2-mediated phosphorylation of RRN3, which regulates repetitive cycles of rDNA transcription initiation [15, 17, 81]. Indeed, we found that CK2-mediated phosphorylation of GST-TCOF1-LCR6 fragment containing one of the repeats promoted its interaction with the purified EGFP-POLR1A *in vitro* (Supplementary Fig. S5G). Furthermore, the TCOF1:POLR1A PLA signal was increased in RPE-PPM1D-KO cells compared to the parental RPE cells, indicating that PPM1D-mediated dephosphorylation is required for proper transcriptional function (Fig. 4D). This dysregulated interaction between TCOF1 and POLR1A in RPE-PPM1D-KO cells was also linked with diminished 5EU incorporation, indicating that PPM1D activity is needed for rDNA transcription (Fig. 4E). Expression of the wild-type PPM1D in RPE-PPM1D-KO cells rescued the abnormal interaction between TCOF1 and POLR1A as well as the level of rDNA transcription, thus confirming the specificity of the phenotype (Fig. 4D and E). To get more insight into how TCOF1 phosphorylation regulates its loading on rDNA and influences RNA Pol I-dependent transcription, we performed ChIP from cells expressing the wild-type TCOF1 or the TCOF1-SA mutant and determined the loading of TCOF1 and Pol I on rDNA using qPCR. We found that the loading of TCOF1-SA mutant to the proximity of the rDNA transcription start was impaired compared to the wild-type TCOF1 (Fig. 4F and Supplementary Fig. S5H). This was connected to the significantly decreased occupancy of RNA Pol I throughout the rDNA in cells expressing TCOF1-SA (Fig. 4F). Inversely, TCOF1 and RNA Pol I loading to the rDNA transcription start was significantly increased in PPM1D-KO cells, in which an increased TCOF1 phosphorylation is expected (Fig. 4G). Interestingly, occupancy of RNA Pol I in the rDNA gene body did not significantly differ in RPE-PPM1D-KO and parental RPE cells, suggesting a potential imbalance between RNA Pol I accumulation at transcription start and its release in the elongation phase (Fig. 4G). Together, these findings demonstrate that reversible phosphorylation of TCOF1, regulated by CK2 and PPM1D, contributes to regulating its interaction with Pol I and to maintaining efficient rDNA transcription. In contrast, we did not find any significant differences in rRNA processing and stability between RPE-PPM1D-KO and parental RPE cells (Supplementary Fig. S5I–K). Similarly, localization of NBS1 in the nucleolar FCs was comparable in parental and RPE-PPM1D-KO cells, suggesting that the reduced rDNA transcription in the absence of PPM1D was not caused by the impaired recruitment of NBS1 (Supplementary Fig. S6A) [82].

Dysregulation of rDNA transcription frequently manifests in altered nucleolar morphology [83]. Indeed, transmission electron microscopy revealed severe defects in organization of the nucleoli in PPM1D-KO cells with indistinguishable FC and DFC components, indicative of impaired rDNA transcription (Fig. 4H). Consistent with earlier observations with shRNA-mediated depletion of PPM1D [84], RPE-PPM1D-KO or U2OS-PPM1D-KO cells displayed enlarged nucleoli, a phenotype that was rescued by re-expression of the wild-type PPM1D but not by the catalytically inactive PPM1D-D314A mutant (Fig. 4I). Increase in nucleolar size was not dependent on p53 activity as it was still apparent in PPM1D-TP53 double knockout cells (Supplementary Fig. S6B). Inversely, expression of the non-phosphorylated EGFP-TCOF1-SA or EGFP-TCOF1-ΔRD in cells lacking the endogenous TCOF1 showed decreased size of the nucleoli, indicating that the phosphorylation status of TCOF1 affects the size of the nucleoli (Fig. 4J). Altogether, phosphorylation of the RD domain of TCOF1 is needed for its interaction with Pol I and for rDNA transcription, while defects in this modification can manifest by impaired nucleolar morphology.

### Inhibition of PPM1D sensitizes cells to RNA polymerase I inhibition

Given the transcriptional defect in PPM1D-KO cells, we hypothesized that loss of PPM1D function may sensitize cells to Pol I inhibition. To explore this, we analyzed data from the genome-wide CRISPR dependency screens using the DepMap portal [85]. Notably, the dependency on the PPM1D gene strongly correlated with the sensitivity to RNA Pol I inhibitors CX-5461 and ActD (Fig. 5A). Next, we analyzed the correlation between PPM1D dependency and drug sensitivity in the cell lines available in the DepMap portal (Fig. 5B). As expected, a p53-stabilizing compound nutlin-3 ranked the highest, consistent with the well-established regulation of p53 response by PPM1D and high sensitivity of cancer cells with the wild-type p53 to PPM1D inhibition [38, 40, 44, 50, 69, 86]. Interestingly, both CX-5461 and ActD also emerged among the top hits, indicating that cells whose proliferation depends on PPM1D may be sensitive to Pol I inhibition (Fig. 5B). Indeed, we observed that the pharmacological inhibition of PPM1D sensitized both RPE and MCF7 cells to CX-5461 and ActD (Fig. 5C). In addition, we found that inhibition of PPM1D promoted activation of p53 and increased the level of cell death induced by ActD as determined by detection of phosphorylated p53 S15 and the cleaved Caspase 7, respectively (Fig. 5D). Nucleolar defects as well as PPM1D inhibition are both known to strongly induce p53 response [38, 86–88]; therefore, we asked to what extent the impact of PPM1D inhibition on proliferation of cells treated with Pol I inhibitors depends on p53 pathway. To determine this, we used RPE-TP53-KO and MCF7-TP53-KO cells and tested their sensitivity to Pol I and PPM1D inhibitors. As anticipated, sensitivity to CX-5461 and ActD was largely p53-dependent (Fig. 5E). Interestingly, the inhibition of PPM1D enhanced the sensitivity of TP53-KO cells to both RNA Pol I inhibitors, suggesting that the effect of PPM1D on cellular proliferation is at least partially p53-independent, possibly reflecting a direct role of PPM1D in the nucleolus (Fig. 5E). Finally, we confirmed that increased sensitivity of RPE-PPM1D-KO cells to Pol I inhibitors can be rescued by inducible expression of EGFP-PPM1D-WT (Fig. 5F). Taken together, these results indicate that loss of PPM1D sensitizes cells to RNA Pol I inhibition, through both p53-dependent and independent mechanisms.

**Figure 5.**
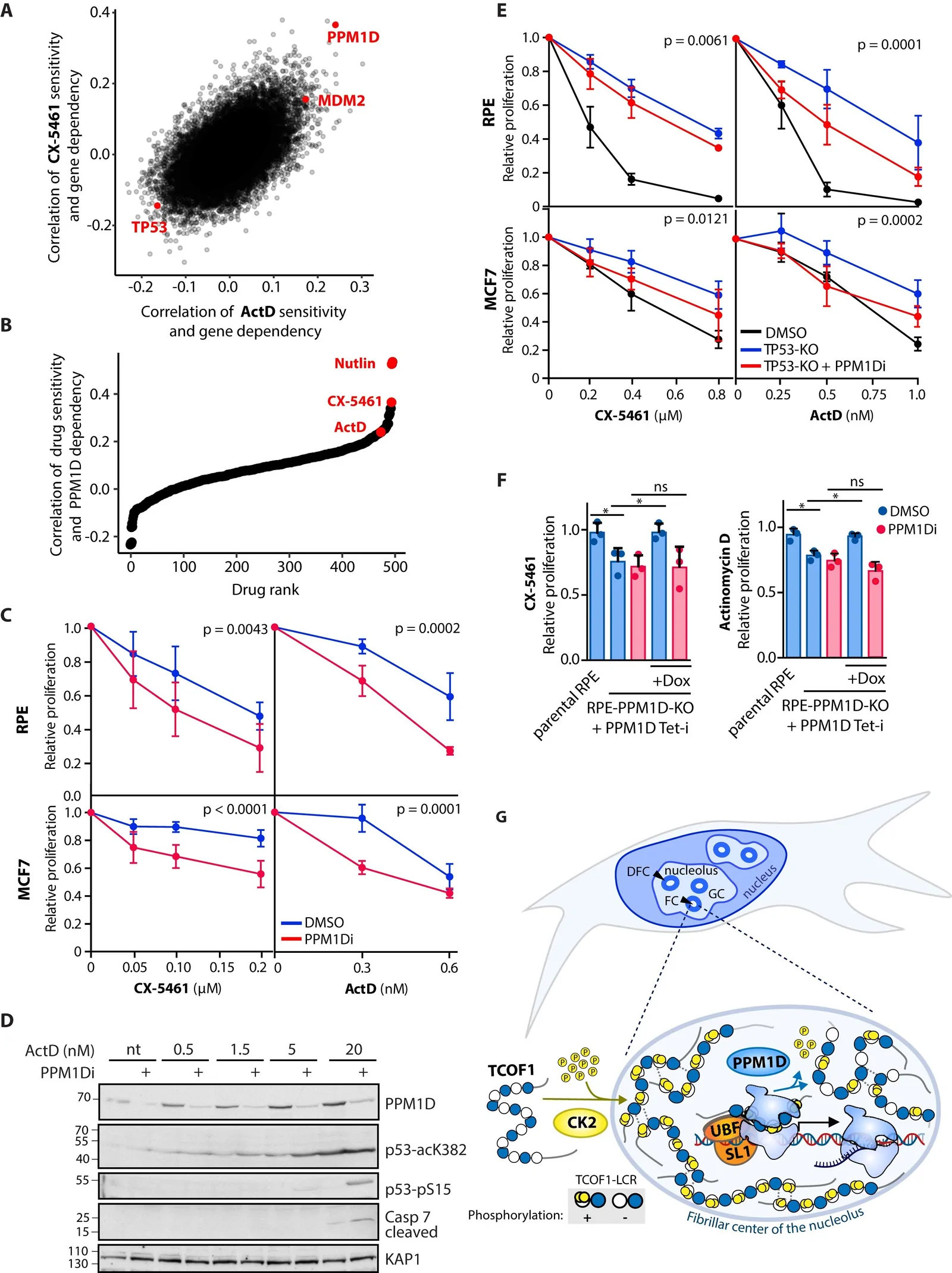
Loss of PPM1D disrupts nucleolar morphology and sensitizes cells to RNA Pol I inhibition. (**A**) Correlation of gene dependency (DepMap-Public-25Q2 + Score-Chronos) and CX-5461 sensitivity (Sanger-GDSC1-AUC) plotted together with correlation for ActD sensitivity (Sanger-GDSC2-AUC), *n* ≥ 221. (**B**) Correlation of PPM1D-dependency (DepMap-Public-25Q2 + Score-Chronos) with drug sensitivity (Combined GDSC1 and GDSC2 datasets), *n* ≥ 220. (**C**) RPE and MCF7 cells were pre-treated with either PPM1Di (0.5 µM) or DMSO. The next day, cells were additionally treated with indicated doses of ActD and CX-5461. The relative proliferation was evaluated using resazurin viability assay after 7 days (mean ± SD, *n* = 3, two-way ANOVA). (**D**) MCF7 cells were pre-treated with DMSO or PPM1Di (1 µM) for 3 h and then treated with indicated dose of ActD for 30 h. The levels of activated p53 and cleaved Caspase 7 were analyzed using immunoblotting. (**E**) Parental RPE, MCF7 cells, and derived TP53-KO cells were pre-treated with either PPM1Di (1 µM) or DMSO. The next day, cells were additionally treated with indicated doses of ActD and CX-5461. The relative proliferation was evaluated using resazurin viability assay after 7 days. Two-way ANOVA was used to evaluate difference in viability of PPM1Di or DMSO treated TP53-KO cells (mean ± SD, *n* = 4). (**F**) RPE-PPM1D-KO cells with inducible EGFP-PPM1D expression were pre-treated with doxycycline and/or PPM1Di (1 µM). The next day, cells were additionally treated with ActD (0.15 nM) and CX-5461 (200 nM). The relative proliferation was evaluated using resazurin viability assay after 7 days (mean ± SD, *n* = 3, two-sample *t*-test, *P < .05, ns *P* ≥ .05). (**G**) Model of regulation of the nucleolar TCOF1 function by phosphorylation. Blue and white circles depict the repeat regions, yellow circles depict the phosphorylation events, and dashed lines indicate interactions between regions with the opposing charges.

## Discussion

PPM1D has a well-established role in timely termination of the DNA damage response and inhibition of the p53 pathway that allows cells to escape the tumor-suppressive barriers and underlies its oncogenic properties [37, 39, 42–45, 50, 86]. In contrast, the checkpoint-independent functions of PPM1D only begin to emerge. Depletion of PPM1D was recently reported to increase the nucleolar size, and PPM1D was suggested to influence nucleolar architecture indirectly through affecting the activity of CDK1 [84]. In this study, we provide evidence that PPM1D regulates nucleolar function by directly targeting the nucleolar substrates. We have identified a NoLS signaling sequence in the unstructured C-terminal domain of PPM1D that promotes its nucleolar localization. By combining biochemistry, PLAs, and super-resolution microscopy, we showed that PPM1D interacts with TCOF1 and can localize to the nucleolar FCs, where it regulates the Pol I-dependent transcription of rDNA. Electron microscopy revealed that loss of PPM1D severely impaired nucleolar architecture and diminished the resolution of FC, DFC, and GC compartments. Formation of the nucleolar compartments depends on the LLPS mediated by homotypic and heterotypic interactions between the nucleolar proteins and rRNA [28, 30, 31, 57]. Reversible protein phosphorylation has recently been identified as an important mechanism for regulating assembly and disassembly of biomolecular condensates [89]. Our phosphoproteomic screen identified TCOF1 and its RD as a direct target of PPM1D that opposes the CK2-mediated phosphorylation. Subsequent analysis revealed that the phosphorylation status of TCOF1 influenced its ability to form biomolecular condensates; thus, the self-assembly of EGFP-TCOF1 was impaired upon CK2 inhibition or PPM1D overexpression, and the same phenotype was observed with the non-phosphorylatable TCOF1-SA variant. On the other hand, we did not detect any defects in NBS1 localization to FC in PPM1D-KO cells, which is a process that is mediated by TCOF1 and has been proposed to facilitate pre-rRNA maturation [82]. This suggests that the nucleolar defects present in PPM1D-KO cells are not caused by impaired NBS1 localization to the nucleoli. Besides TCOF1, NPM1 undergoes LLPS in the nucleolar GC compartment, and its phosphorylation at S254 and S260 residues was recently implicated in inhibition of this process [31, 89]. In our screen, we did not detect any enrichment of NPM1 phosphopeptides in PPM1D-KO cells, preventing us from testing if PPM1D could partly regulate nucleolar functions through targeting NPM1. Our data support a model in which phosphorylation of TCOF1 directly facilitates binding of RNA Pol I and its loading to rDNA. Cells lacking PPM1D showed increased loading of RNA Pol I to rDNA transcription start, likely reflecting an increased modification of TCOF1, but the rDNA transcription was decreased. We propose a model according to which the CK2-mediated phosphorylation of TCOF1 enhances its ability to form condensates and recruit Pol I, while PPM1D-dependent dephosphorylation may be required to allow proper handoff or release during transcription cycles (Fig. 5G). This mechanism bears striking similarity to the regulation of RRN3, another Pol I-associated factor, which is released from the transcription complex upon CK2 phosphorylation, and subsequent dephosphorylation by FCP1 is required for renewing the interaction necessary for continuous transcription [16, 17]. Unlike the general initiation factors SL1 or RRN3, which are indispensable for promoter engagement, TCOF1 appears to function as a nucleolar scaffold whose reversible phosphorylation fine-tunes Pol I recruitment and dynamic release [90]. In this study, we addressed the impact of opposing activities of CK2 and PPM1D on nucleolar functions of TCOF1. Several nucleolar proteins, including TCOF1, have recently been reported to undergo extensive modification by inositol pyrophosphates, and it will be interesting to test if CK2 and PPM1D may indirectly influence the level of TCOF1 pyrophosphorylation [91]. Of note, the rate of rDNA transcription has previously been associated with nucleolar size, with actively transcribing cells (e.g. cancer cells) exhibiting larger nucleoli [92]. Enlargement of the nucleoli can also result from the accumulation of pre-rRNA processing intermediates. Nevertheless, since we did not detect any significant differences in rRNA processing in cells lacking PPM1D, we favor the possibility that the increased nucleolar size is caused by enhanced self-assembly of TCOF1 rather than by accumulation of rRNA intermediates. On the other hand, due to the observed interaction between PPM1D and FBL, we currently cannot rule out some contribution of PPM1D to rRNA processing.

TCOF1 is thought to originate from a duplication event of the *NOLC1* gene during the evolution of tetrapods, which coincides with the appearance of a defined nucleolar FC [33]. Interestingly, the conserved NoLS of PPM1D also first appears in tetrapods, suggesting a coordinated evolutionary development of both structural and regulatory components of the nucleolus. Besides TCOF1, we noted enrichment of phosphorylation of several other proteins with LLPS potential in cells lacking PPM1D, including the TCOF1 orthologue NOLC1 (also known as Nopp140). Although we have not yet explored their functional relevance, these observations point to a broader role for PPM1D in regulating the composition and dynamics of biomolecular condensates. Accordingly, a recent phosphoproteomic screen identified that PPM1D regulates etoposide-induced phosphorylation of several proteins with phase separation capabilities (including SRRM2, ACIN1, CREBBP, and PABPN1) but these targets have thus far not been functionally tested [46]. Interestingly, PPM1D itself contains IDRs, including the proline-rich loop and C-terminal domain; although poorly conserved, they are present throughout the species. This raises the possibility that PPM1D could associate with condensates through the heterotypic interactions. A similar model has recently been suggested for Casein kinase 1 delta, in which the IDR facilitates the binding to the condensates of its substrate, TDP-43 [93].

It is well established that impaired ribosome biogenesis triggers the p53 response through accumulation of RPL11/RPL5-5S rRNA complex in the nucleoplasm, leading to inactivation of MDM2 and stabilization of the p53 [83, 87, 88, 94]. The inhibition of rDNA transcription has been proposed as a promising strategy for cancer treatment, and several inhibitors of Pol I entered clinical trials [95, 96]. While CX-5461 was developed as a Pol I transcription initiation inhibitor, recent findings show that it also acts as a Top2α inhibitor that preferentially induces double-strand breaks at the rDNA promoter and 5′ETS regions [97]. Although our data indicates that PPM1D is not required for rDNA break repair (Supplementary Fig. S2), PPM1D deficiency robustly sensitizes cells to CX-5461. Crucially, a similar sensitization was observed with low doses of ActD, which suppresses Pol I transcription without generating Top2α-dependent rDNA breaks. This suggests that the lethality observed upon PPM1D inhibition is predominantly mediated by Pol I transcriptional arrest and nucleolar perturbation rather than defective rDNA damage repair. Among a large number of various cancer cell lines analyzed by DepMap portal, we noted a strong correlation between the dependency on PPM1D and sensitivity to two Pol I inhibitors, suggesting that cancer cells might be highly sensitive to the combined inhibition of PPM1D and Pol I. Not surprisingly, we find that the sensitivity of cells to Pol I inhibitors is by a large part mediated by p53 pathway. Sensitivity to the combined inhibition of PPM1D and Pol I will likely be mediated by an increased stimulation of p53 pathway by increased phosphorylation of p53, its acetylation, and stabilization by inhibition of MDM2. Nevertheless, at least part of the sensitivity to PPM1D inhibition is mediated by p53-independent mechanisms, possibly reflecting the nucleolar function of PPM1D. Potential exploitation of the combined inhibition of PPM1D and Pol I for suppressing the tumor growth is an attractive topic for future research.

In summary, our data supports a model in which PPM1D and CK2 regulate the condensation and function of TCOF1 through the dynamic phosphorylation of its RD domain. This regulation controls TCOF1’s association with Pol I and its role in rDNA transcription. This finding expands our understanding of how phosphatases shape nuclear compartmentalization and suggests that PPM1D may act as a broader regulator of LLPS through selective targeting of LCRs.

## Supplementary Material

gkag1060_Supplemental_Files

## Data Availability

Data from the mass spectrometry analysis were uploaded to the ProteomeXchange Consortium via the PRIDE repository (https://www.ebi.ac.uk/pride/archive) with the dataset identifier (PXD065997).
