# Supplementary material for: Dynamic phosphorylation of TCOF1 controlled by CK2 and PPM1D regulates nucleolar condensation and RNA polymerase I activity
Source: Nucleic Acids Res. 2026 Oct 30;54(20):gkag1060. doi: 10.1093/nar/gkag1060 (PMC13720506; doi:10.1093/nar/gkag1060)
Supplement: Supplementary file 1 [file gkag1060_supplemental_files.zip › SUPPLEMENTARY FIGURES-REV2-combined-FINAL.pdf]

SUPPLEMENTARY DATA

Dynamic phosphorylation of TCOF1 controlled by CK2 and PPM1D regulates nucleolar condensation and RNA Polymerase I activity

Natalie Palkova[1,#], Matous Palek[1,#], Marketa Brodinova[1], Zuzana Chalupova[1], Radka Storchova[1], Jana Jasprova[2], Pavel Talacko[2], Dusan Cmarko[3], Libor Macurek[1,*]

[1] *Cancer Cell Biology, Institute of Molecular Genetics of the Czech Academy of Sciences, Prague, CZ-14220, Czech Republic*

[2] *BIOCEV Proteomics Core Facility, Faculty of Science, Charles University, Vestec, 252 50, Czech Republic.*

[3] *Institute of Biology and Medical Genetics, First Faculty of Medicine, Charles University and General University Hospital in Prague, 128 01 Prague, Czech Republic.*

SUPPLEMENTARY FIGURE LEGENDS

Supplementary Figure 1. PPM1D localization in the nucleolus

Supplementary Figure 2. PPM1D is not essential for repair of ribosomal DNA

Supplementary Figure 3. Phosphopeptides regulated by PPM1D

Supplementary Figure 4. FRAP microscopy of TCOF1 variants

Supplementary Figure 5. Impact of PPM1D on nucleolar functions

Supplementary Figure 6. PPM1D impacts nucleolar functions independent of p53

Supplementary Table 1. Phosphoproteomic analysis of U2OS cells under basal conditions, following DNA damage and after PPM1D modulation.

Supplementary Table 2. List of seeds used for AlphaFold3 predictions.

Supplementary Table 3. Sequence of primers used for ChIP-qPCR analysis

Supplementary Figure 1. PPM1D localization in the nucleolus

A. MCF7 cells were stained with PPM1D and PPM1G antibodies and imaged by confocal microscopy. A representative single deconvolved planes are shown. The scale bar represents 10 μm.

B. Sequence alignment of PPM1D from different species. The conserved residues are highlighted in red, the residues in the NoLS region in yellow.

C. 3D-SIM imaging of MCF7 cells stained with PPM1D and POLR1A antibody (scale bars 10 μm and 1 μm). Shown are the deconvolved single plane and an enlarged nucleolar region with XZ and YZ orthogonal sections indicated by dashed lines.

Supplementary Figure 1

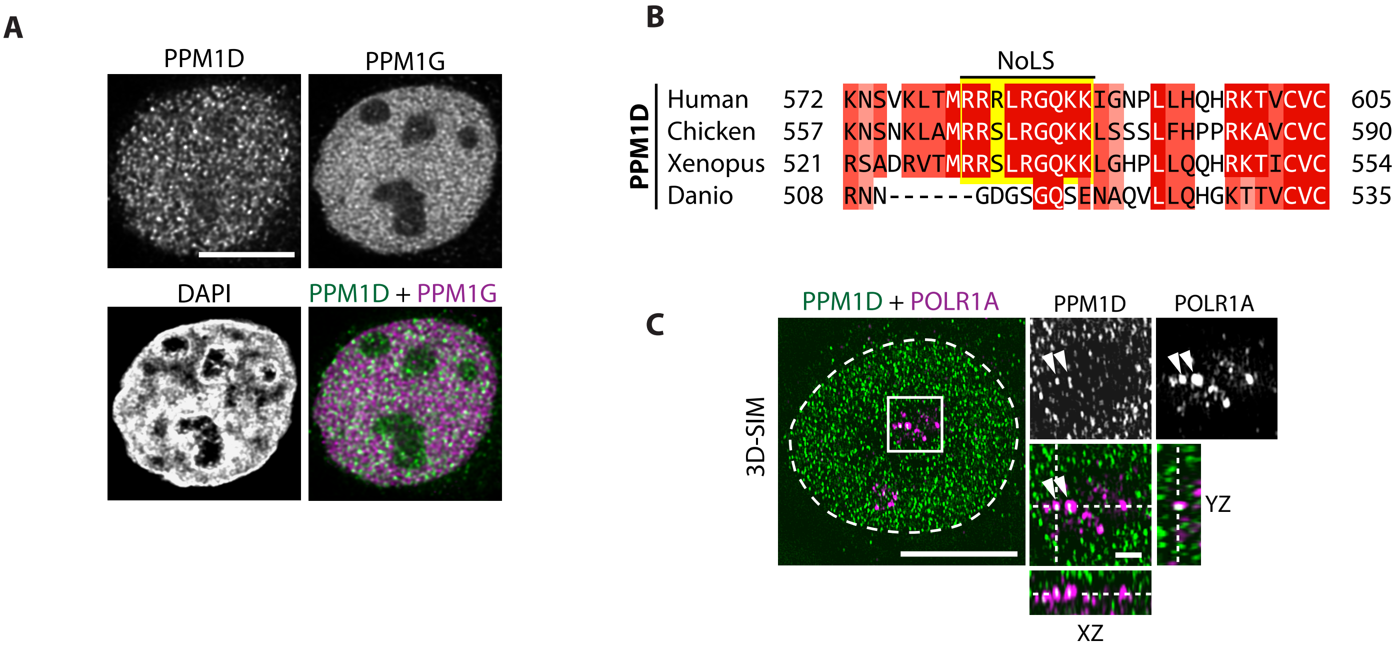

Supplementary Figure 2. PPM1D is not essential for repair of ribosomal DNA

A. RPE-iCut cells were treated overnight with Shield-1 and doxycycline to induce expression of Cas9 endonuclease that was targeted to DNA upon transfection of sgRNA targeting 45S rDNA repeats. Where indicated, cells were pre-treated overnight with PPM1Di (1 μM). Cells were fixed 8h after sgRNA transfection and stained for UBF. The scale bar represents 10 μm. The mean nucleolar caps count is shown ±SD is shown from representative experiment (n=300), Mann–Whitney test (ns P≥0.05).
B. RPE-iCut cells were treated as in A, fixed at indicated time points and stained for γH2AX. The mean γH2AX intensity is shown ±SD is shown from representative experiment (n=400), Mann–Whitney test (****P <0.0001).
C. RPE-iCut cells were treated as in A, fixed at indicated time points and stained for RAD51. The mean RAD51 foci count is shown ±SD from representative experiment (n≥179), Mann–Whitney test (ns P≥0.05). Representative images are shown, the scale bar corresponds to 10 μm.
D. RPE1-iCut cells were treated overnight with doxycycline and Shield-1 before transfection of sgRNA targeting 45S rDNA. Cells were pretreated overnight with 1μM PPM1D inhibitor or DMSO as indicated. Relative proliferation was determined by resazurin assay after 7 days and was normalized to non-treated cells (n=4), two-way ANOVA.

Supplementary Figure 2

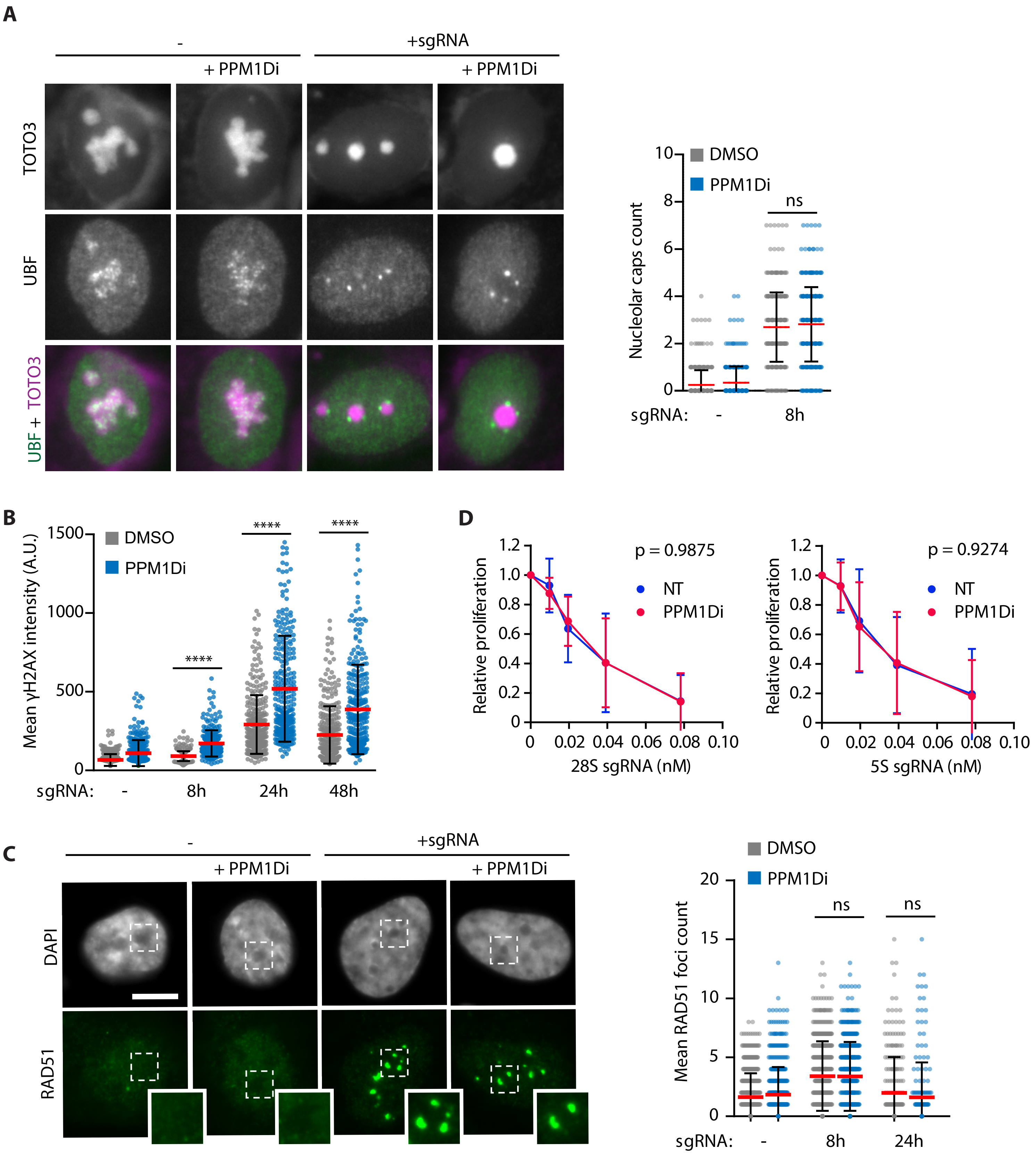

Supplementary Figure 3. Phosphopeptides regulated by PPM1D

A. Titration of PPM1D inhibitor. Parental U2OS and U2OS-PPM1D-KO cells were treated with indicated doses of GSK2830371 for 2 h and whole cell lysates were probed with indicated antibodies. Note expected increase of the pSer824-KAP1 and pT68-CHK2 signal in parental U2OS cells and no effect of inhibitor treatment in U2OS-PPM1D-KO cells.

B. Sequence logo analysis of etoposide induced phosphorylations regulated by PPM1D (n=123) and non-regulated by PPM1D (n=878).

C. Sequence logo analysis of phosphorylations regulated by PPM1D (n=739) and non-regulated by PPM1D (n=5455).

D. GO Biological Process term analysis of PPM1D-regulated and Etoposide-induced phosphorylations using ViseaGO R package. The 12 most enriched terms are shown, p-value was calculated using Fisher exact test.

E. Kinase activities predicted using the Fisher binary enrichment analysis in Kinase Library. Only kinases with nuclear localization and more than 15 substrates are shown, p-value was calculated using Fisher exact test.

F. Dot plot per-residue matrix of the TCOF1 protein sequence generated by EMBOSS Dotmatcher. Altogether, 17 low-complexity regions (appear as black stripes outside the diagonal) were identified and are highlighted in yellow. A schematic of the TCOF1 and its ΔRD and SA variants used in this study is shown below. The number of serine residues mutated to alanine in each individual LCR of the TCOF1-SA mutant is indicated at the bottom.

G. Expression of EGFP-TCOF1 in U2OS cells was induced with overnight doxycycline treatment. Cells were then either mock-treated or treated with CK2i for 3 h. Cell extracts supplemented with phosphatase inhibitors were incubated with GFP trap. Proteins were separated by SDS-PAGE on 5% gel. Shown is representative result from 4 independent experiments. Note the mobility shift in condition with CK2i.

H. Cells were treated as in G and the separation was performed using a gradient 5-20% gel. EGFP-TCOF1 phosphorylation was detected using immunoblotting with phospho-CK2-motif antibody.

I. Testing specificity of TCOF1 antibody in immunoblotting. Cells transfected with control siRNA or siRNA to TCOF1 were collected after 48 h. Whole cell lysates were analyzed by immunoblotting. A representative result is shown. Signal of TCOF1 was normalized to the signal of histone H3 and was quantified from three independent experiments using ImageJ.

Supplementary Fig. 3

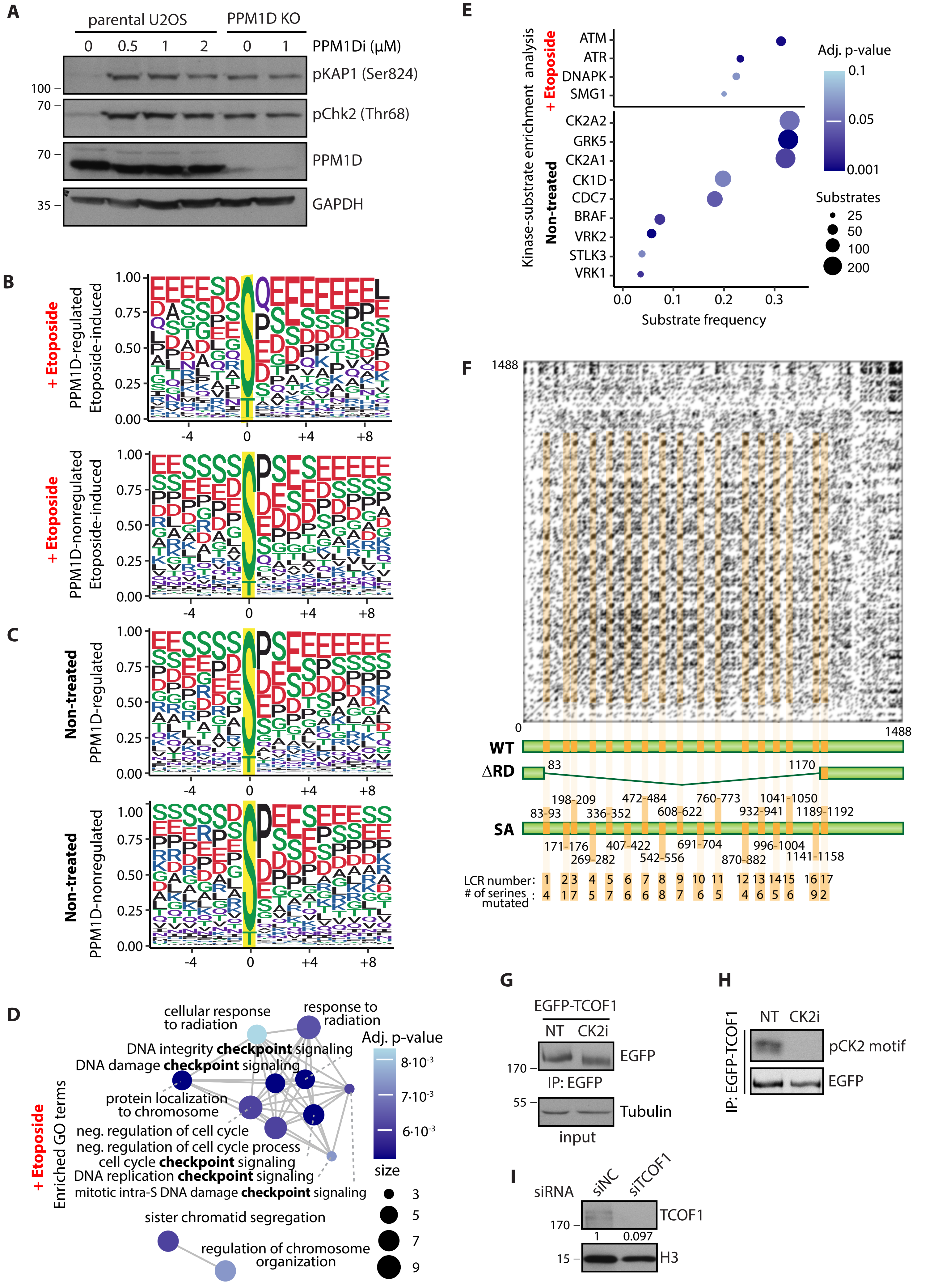

Supplementary Figure 4. FRAP microscopy of TCOF1 variants

A. Scheme describing endogenous tagging of PPM1D in RPE cells. Diagnostic PCR from genomic DNA and DNA fragments that were validated by Sanger sequencing are shown below.

B. Representative figures from Fig. 2M. Cells were transfected with EGFP-PPM1D-WT or -ΔNoLS and PLA reactions were performed using antibodies to GFP and TCOF1. Cells were gated according to EGFP signal into positive and negative groups.

C. Expression of the wild-type EGFP-TCOF1, SA and ΔRD variants was induced by overnight doxycycline treatment. Cells were then stained with TOTO-3. The scale bar corresponds to 10 μm.

D. Expression of the EGFP-TCOF1-WT was induced for 6 h with doxycycline. Where indicated, cells were transfected with plasmid coding for FLAG-PPM1D or were treated with CK2i 3 h prior fixation. Cells were then stained with TOTO-3 and anti-FLAG antibody. The scale bar corresponds to 10 μm.

E. Expression of wild-type EGFP-TCOF1, SA and ΔRD variants was induced by overnight doxycycline treatment. TCOF1 mobility was analyzed by FRAP. The mean signal intensity in the regions of interest is plotted ±SE from three pooled replicate experiments, n≥28. Arrows indicate the immobile fractions of individual TCOF1 variants. Representative images of the identical fields prior to the bleaching, at the bleaching and 40 s after bleaching are shown, dashed lines indicate the bleached regions, the scale bar corresponds to 5 μm.

F. Expression of the EGFP-TCOF1-WT was induced for 6 h with doxycycline and where indicated, CK2i was added for last 3 h. TCOF1 mobility was analyzed by FRAP. The mean is plotted ±SE from three pooled replicate experiments, n≥28.

G. Parental U2OS, U2OS-PPM1D-KO and U2OS-PPM1D-KO cells stably expressing FLAG-PPM1D WT or D314A mutant were treated overnight with doxycycline to induce EGFP-TCOF1-WT expression. TCOF1 mobility was analyzed by FRAP. The mean is plotted ±SE from three pooled replicate experiments, n≥29.

Supplementary Fig. 4

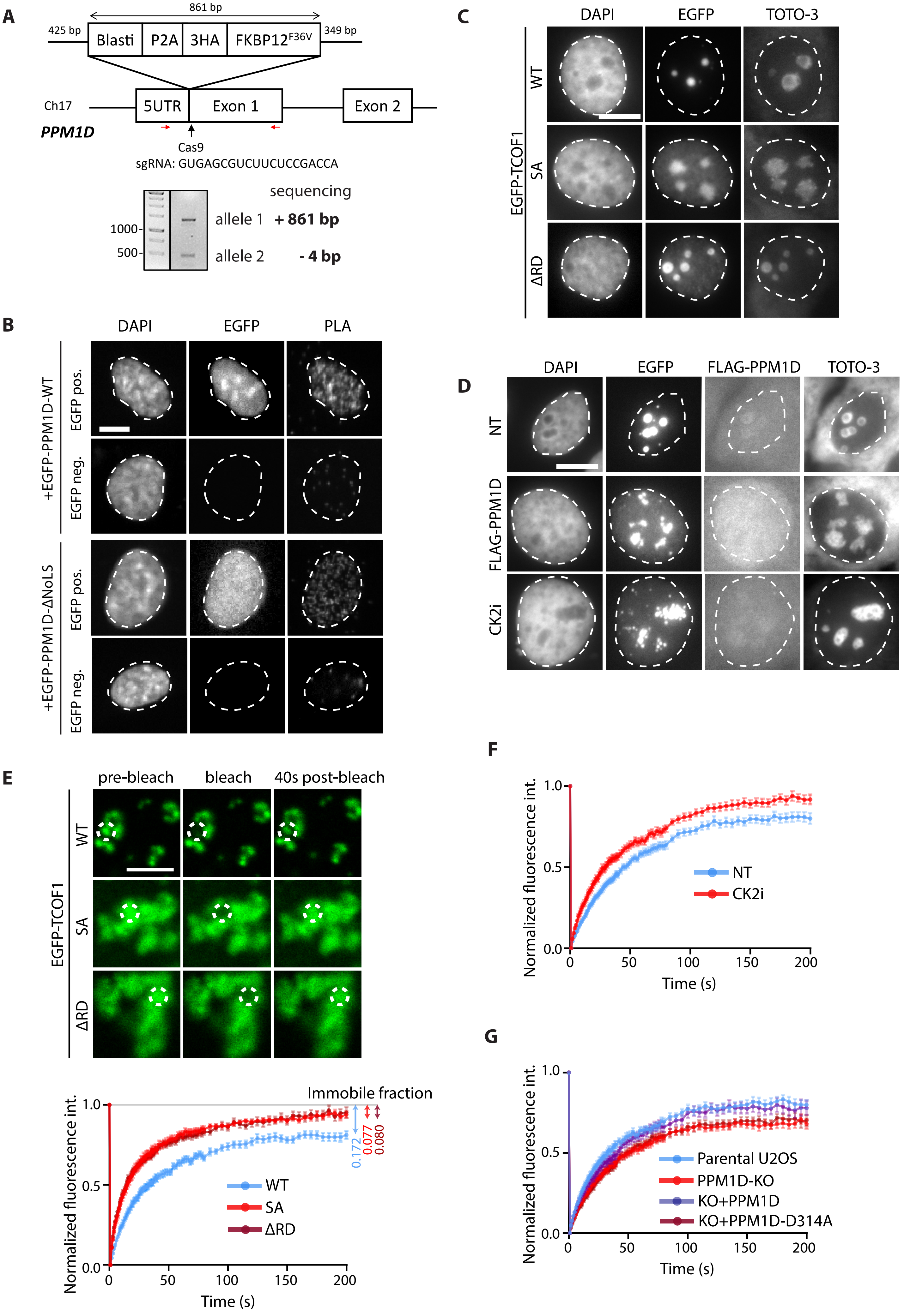

Supplementary Figure 5. Impact of PPM1D on nucleolar functions

A. Cells expressing EGFP, EGFP-TCOF1 or EGFP-TCOF1-SA were extracted and incubated with GFP trap. After washing, bound proteins were analyzed by immunoblotting. Numbers indicate signal intensity normalized to the amount of purified TCOF1 and to the wild-type condition. Representative experiment from 2 repeats.

B. Cells expressing the wild-type EGFP-TCOF1, -SA or -ΔRD mutants were fixed and PLA between EGFP and UBF was analyzed. Three independent repeats, two-tailed t-test (*P <0.05, **P <0.01).

C. Cells were depleted for endogenous TCOF1 for 4 days by repeated transfection of siRNA. Expression of siRNA resistant wild-type EGFP-TCOF1, SA and ΔRD variants was induced by overnight treatment with doxycycline. TCOF1 levels were evaluated using immunoblotting.

D. Cells from Fig. 4B were scored for the levels of nuclear EGFP-TCOF1. No significant differences between the wild type TCOF1 and, -SA or -ΔRD mutants were found.

E. Predicted aligned error matrix for prediction of interaction of TCOF1 with POLR1A and POLR1B. Prediction for non-phosphorylated TCOF1 is shown on the left, for phosphorylated TCOF1 at repeats 16,17 on the right.

F. Interaction template modelling score (iPTM) for interaction between POLR1A and TCOF1 phosphorylated at different repeat pairs. Position of repeat pairs that are phosphorylated are indicated in a scheme of the primary structure of TCOF1. Plotted are mean iPTM ±SD from 5 predictions generated with different seeds. Statistical significance was evaluated using two-tailed t-test (*P <0.05, ****P <0.0001).

G. Recombinant GST or GST-TCOF1-LCR6 was incubated with CK2 in the presence or absence of ATP for 20 min as indicated and subsequently incubated with EGFP-POLR1A immunopurified from transfected cells using GFP-Trap. After extensive washing, bound proteins were analyzed by immunoblotting. A representative experiment from three independent repeats is shown.

H. Schematic diagram of the rDNA gene with indicated positions (arrowheads) of primers used for ChIP-qPCR analysis. Sequences of primers are shown in Supplementary Table 2.

I. Schematic diagram of the ITS1 and ITS2 probes used for northern blotting analysis in J.

J. Processing of rDNA was determined by northern blot analysis in parental RPE and RPE-PPM1D-KO cells. Quantification of signal intensity of individual rDNA fragments was performed from 4 independent experiments and was normalized to the input control. Plotted is the ratio of values from PPM1D-KO and parental cells.

K. Parental RPE and RPE-PPM1D-KO were treated with 1 mM 5EU for 1 h and then were chased with 5 mM uridine for 0, 1 and 2 h. Mean relative nucleolar 5EU intensity was determined by high-content microscopy (n=3).

Supplementary Fig. 5

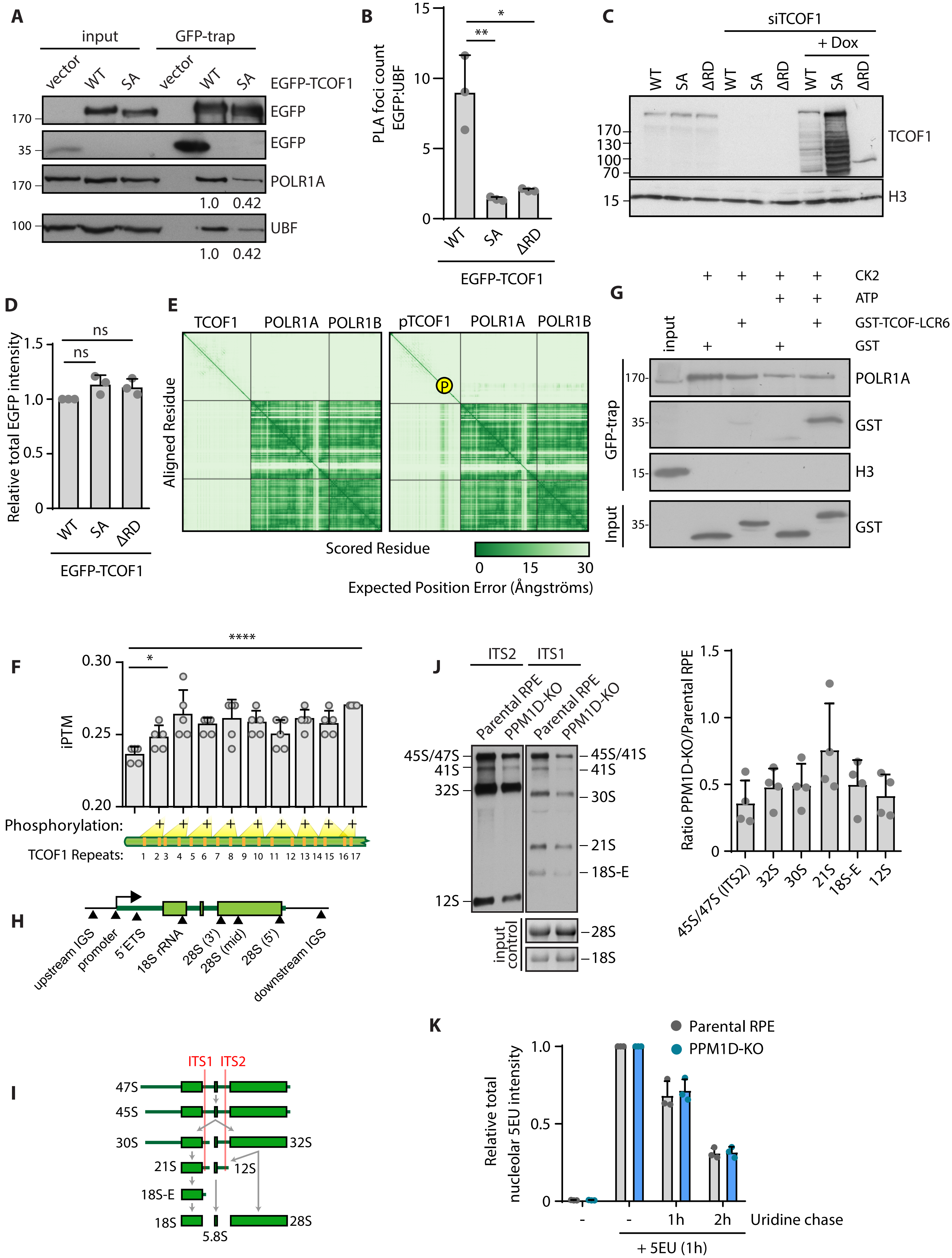

Supplementary Figure 6. PPM1D impacts nucleolar functions independent of p53

A. Parental U2OS and PPM1D-KO cells were IR-irradiated (3Gy) and fixed at indicated timepoints. Cells were then stained for NBS1 and TCOF1 and analyzed by high-content microscopy. The fibrillar center area was segmented based on TCOF1 signal using the spot detection module in ScanR analysis software. Shown is the relative mean intensity of NBS1 in fibrillar center area divided by mean nuclear NBS1 intensity. Representative experiment is shown from two independent repeats (mean ±SD, n=300, Mann–Whitney test, ns P≥0.05).

B. Parental U2OS, PPM1D-KO and PPM1D-TP53-KO cells were fixed and stained for fibrillarin and the mean nucleolar area was analyzed using high-content microscopy. Representative experiment is shown from two independent repeats (mean ±SD, n=300, Mann–Whitney test, **** P<0.0001). The scale bar in the representative images corresponds to 10 μm.

Supplementary Fig. 6

A

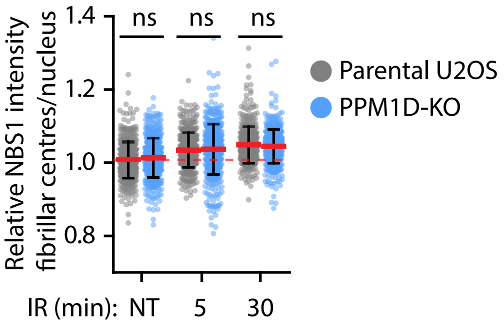

B

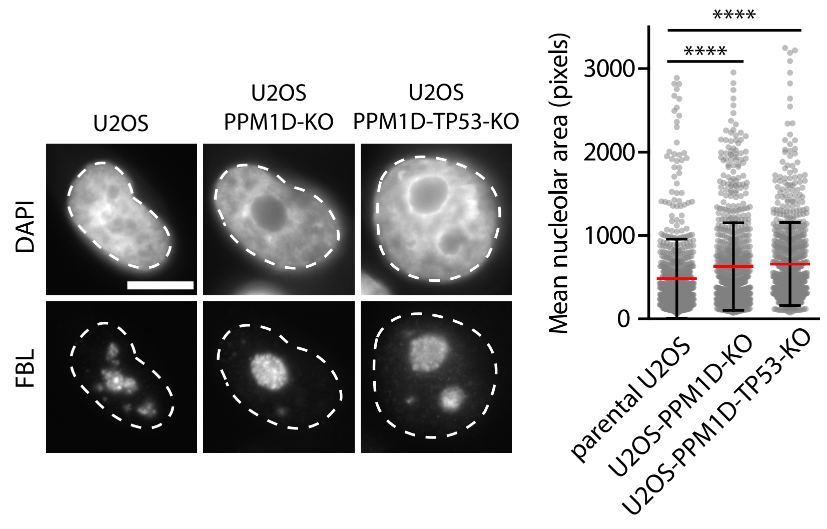

Supplementary Table 1. Phosphoproteomic analysis of U2OS cells under basal conditions, following DNA damage and after PPM1D modulation.

Summary of phosphoproteomic screen results performed in triplicate. U2OS wild-type (WT) and PPM1D-knockout (PPM1D-KO) cells were treated with Etoposide (ETO) or left non-treated (NT). Additionally, WT cells were treated with a PPM1D inhibitor (PPM1Di) in the presence or absence of Etoposide. Columns description: protein / protein_name / gene: Uniprot accessions, protein descriptions, and official gene symbols. position: Amino acid position of the phosphorylated residue within the protein sequence. seq: Flanking peptide sequence (31 amino acids) centered on the phosphorylation site. site: Unique identifier for each phosphosite (Gene_Position). log2 difference: The difference in mean log2-transformed Label-Free Quantification (LFQ) intensities between the two indicated conditions. This value represents the log2 fold-change in phosphopeptide abundance. Positive values indicate increased phosphorylation, negative values indicate decreased phosphorylation (dephosphorylation). -log(P): Statistical significance expressed as the negative log10 of the p-value calculated from two-tailed t-test. Values above 1.3 corresponds to $p < 0.05$. ETO - NT: Change in phosphorylation in WT cells in response to Etoposide. PPM1D-KO - NT / PPM1Di - NT: Baseline differences in phosphorylation caused by the loss or inhibition of PPM1D. PPM1D-KO+ETO - ETO / PPM1Di+ETO - ETO: Comparison of the Etoposide-induced response in the absence of PPM1D activity relative to the response in WT cells. These columns identify sites where PPM1D activity normally modulates the DNA damage response. enriched_term_rRNA_processes: Sites marked with "YES" indicate proteins belonging to the significantly enriched GO term "rRNA metabolic process".

Supplementary Table 2. List of seeds used for AlphaFold3 predictions.

File contains a list of seeds used for AlphaFold3 predictions.

Supplementary Table 3. Sequence of primers used for ChIP-qPCR analysis

The table contains sequences of forward and reverse primers used for ChIP-qPCR analysis.
